# Transdiagnostic clustering of self-schema from self-referential judgements identifies subtypes of healthy personality and depression

**DOI:** 10.3389/fninf.2023.1244347

**Published:** 2024-01-11

**Authors:** Geoffrey Chern-Yee Tan, Ziying Wang, Ethel Siew Ee Tan, Rachel Jing Min Ong, Pei En Ooi, Danan Lee, Nikita Rane, Sheryl Yu Xuan Tey, Si Ying Chua, Nicole Goh, Glynis Weibin Lam, Atlanta Chakraborty, Anthony Khye Loong Yew, Sin Kee Ong, Jin Lin Kee, Xin Ying Lim, Nawal Hashim, Sharon Huixian Lu, Michael Meany, Serenella Tolomeo, Christopher Lee Asplund, Hong Ming Tan, Jussi Keppo

**Affiliations:** ^1^Institute of Mental Health, Singapore, Singapore; ^2^Yale-NUS College, Singapore, Singapore; ^3^Faculty of Social Sciences, National University of Singapore, Singapore, Singapore; ^4^School of Biological Sciences, National Technological University, Singapore, Singapore; ^5^KK Women’s and Children’s Hospital, Singapore, Singapore; ^6^Institute of Operations Research and Analytics, National University of Singapore, Singapore, Singapore; ^7^NHG Polyclinics, Singapore, Singapore; ^8^Ministry of Education, Singapore, Singapore; ^9^Singapore Institute for Clinical Sciences, A*STAR, Singapore, Singapore; ^10^Institute of High Performance Computing (IHPC), Agency for Science, Technology and Research (A*STAR), Singapore, Singapore

**Keywords:** self-schema, self-concept, self-referential processing, personality, depression, depression subtypes, clustering

## Abstract

**Introduction:**

The heterogeneity of depressive and anxiety disorders complicates clinical management as it may account for differences in trajectory and treatment response. Self-schemas, which can be determined by Self-Referential Judgements (SRJs), are heterogeneous yet stable. SRJs have been used to characterize personality in the general population and shown to be prognostic in depressive and anxiety disorders.

**Methods:**

In this study, we used SRJs from a Self-Referential Encoding Task (SRET) to identify clusters from a clinical sample of 119 patients recruited from the Institute of Mental Health presenting with depressive or anxiety symptoms and a non-clinical sample of 115 healthy adults. The generated clusters were examined in terms of most endorsed words, cross-sample correspondence, association with depressive symptoms and the Depressive Experiences Questionnaire and diagnostic category.

**Results:**

We identify a 5-cluster solution in each sample and a 7-cluster solution in the combined sample. When perturbed, metrics such as optimum cluster number, criterion value, likelihood, DBI and CHI remained stable and cluster centers appeared stable when using BIC or ICL as criteria. Top endorsed words in clusters were meaningful across theoretical frameworks from personality, psychodynamic concepts of relatedness and self-definition, and valence in self-referential processing. The clinical clusters were labeled “Neurotic” (C1), “Extraverted” (C2), “Anxious to please” (C3), “Self-critical” (C4), “Conscientious” (C5). The non-clinical clusters were labeled “Self-confident” (N1), “Low endorsement” (N2), “Non-neurotic” (N3), “Neurotic” (N4), “High endorsement” (N5). The combined clusters were labeled “Self-confident” (NC1), “Externalising” (NC2), “Neurotic” (NC3), “Secure” (NC4), “Low endorsement” (NC5), “High endorsement” (NC6), “Self-critical” (NC7). Cluster differences were observed in endorsement of positive and negative words, latency biases, recall biases, depressive symptoms, frequency of depressive disorders and self-criticism.

**Discussion:**

Overall, clusters endorsing more negative words tended to endorse fewer positive words, showed more negative biases in reaction time and negative recall bias, reported more severe depressive symptoms and a higher frequency of depressive disorders and more self-criticism in the clinical population. SRJ-based clustering represents a novel transdiagnostic framework for subgrouping patients with depressive and anxiety symptoms that may support the future translation of the science of self-referential processing, personality and psychodynamic concepts of self-definition to clinical applications.

## Introduction

Depression is one of the most prevalent mental disorders worldwide (Bromet et al., 2011), as well as a leading cause of disability (World Health Organization, 2017). However, depression is heterogeneous, with substantial variability in causes, symptomatology, and course of development (Rush, 2007; Goldberg, 2011; Ulbricht et al., 2018). Due to such heterogeneity, depressed individuals may exhibit differing responses to treatments such as psychotherapy. A meta-analysis showed that a large proportion of depressed individuals were non-responsive to psychotherapy (intention-to-treat remission rate ranging between 32 to 37% depending on the severity of depression (De Maat et al., 2007)). Further, a high drop-out rate from psychotherapy schemes among depressive outpatients (17.5% in Cooper and Conklin, 2015; 24.6% in Hans and Hiller, 2013) can compromise treatment efficacy. As such, it is important to better understand the heterogeneity of depression in order to personalize treatment.

According to cognitive models of depression, the presence of negatively-focused self-schemas and negative self-referential processing biases is central to the onset, maintenance, and recurrence of clinical depression (Beck, 1967; Ingram et al., 1983; Scher et al., 2005). Self-referential processing (SRP) refers to the processing of information as related to one’s self (Northoff et al., 2006). Incoming information is remembered best when it is encoded with reference to one’s self as compared to other-reference, semantic, phonemic, and structural encoding (Rogers et al., 1979; Symons and Johnson, 1997; Bentley et al., 2017). This supports the notion that one’s self-concepts serve as an important framework for the encoding, processing, interpretation, and storage of incoming information, which is termed the self-reference effect (Rogers et al., 1979).

Notably, self-schemas implicated in depression are usually characterized by themes of loss, failure, worthlessness, rejection, and hopelessness (Phillips et al., 2010). Such negatively-focused self-schemas often lead to biases in processing self-referential information. Individuals tend to prioritize the encoding and retention of negative self-concepts, thus reinforcing depressive cognitive patterns (LeMoult and Gotlib, 2019). For example, Disner et al. (2017) found that the valence of a person’s self-referential schema significantly predicted the severity of their onset depressive symptoms. Specifically, having a stronger negative self-schema, as opposed to a positive one, was associated with more severe depressive symptoms. Moreover, Dozois (2007) highlighted the stability of the structure of these negative self-schemas over time. Interestingly, the self-schemas often remain stable, even when individuals experience improvements in their depressive symptoms. Similarly, in a longitudinal study with pregnant women conducted by Evans et al. (2005), the authors found that the association between negative self-schemas remained significant and predicted the onset of depression more than 3 years later. This supports the notion that negative self-schemas represent a long-lasting vulnerability to depression.

It is within this framework that the use of self-referential judgements (SRJs) becomes particularly relevant as it serves as an explicit manifestation of these negative self-schemas. By analyzing the content and frequency of SRJs made by individuals, we gain deeper insights into how negative self-concepts are constructed and perpetuated, shedding light on the relationship between SRJs, self-schemas and depression.

The Self-Referential Encoding Task (SRET; Derry and Kuiper, 1981) is a key measure of biases in self-schemas and self-referential processing. In the SRET, participants are asked to make binary decisions about whether positive and negative adjectives describe themselves or not (the endorsement phase), after which they go through a distractor task, and then complete an incidental free recall for those same adjectives (the recall phase). Biases in self-schemas are indicated by the number of positive/negative words that people endorse; biases in SRP are usually assessed by their speed of endorsement or rejection, as well as subsequent recall for positive and negative endorsed words.

Negative and positive biases in the endorsement and recall of negative self-relevant stimuli in the SRET are associated with depression and depressive symptoms (Derry and Kuiper, 1981; Gotlib et al., 2004; Auerbach et al., 2015; Goldstein et al., 2015; Connolly et al., 2016). In the original paper that developed SRET for accessing self-schemas in clinical depression, Derry and Kuiper (1981) found that as compared to non-depressed psychiatric control and healthy control participants, clinically depressed participants showed superior recall for depressive/negative (rather than non-depressive/positive) adjectives endorsed as self-descriptive. Subsequent research demonstrated similar patterns of results across age samples: Gotlib et al. (2004) found that adult patients with major depressive disorder (MDD) endorsed more negative words and fewer positive words as self-descriptive, and recalled a higher proportion of negative endorsed words and lower proportion of positive endorsed words than psychiatric control and healthy control participants. Similar results on another adult sample were obtained by Fritzsche et al. (2010). Likewise, Auerbach et al. (2015) demonstrated that depressed adolescents endorsed more negative and fewer positive words, as well as recalled fewer positive words compared to healthy controls. Goldstein et al. (2015) found that depressive symptoms were positively correlated with the proportion of negative self-referent words recalled, and negatively correlated with the proportion of positive self-referent words recalled, among a community of children at age 6. Similarly, among a community sample of 12-year-old adolescents, depressive symptoms were correlated with higher endorsement of negative words, lower endorsement of positive words, slower RT in rejecting negative words as self-descriptive, as well as higher recall of negative self-referent words and lower recall of positive self-referent words (Connolly et al., 2016). In an adult sample with elevated depressive symptoms, the number of positive and negative words endorsed, negatively and positively predicted baseline depressive symptoms, respectively (Disner et al., 2017). Lastly, using a best subset regression approach, Dainer-Best et al. (2018) discovered that the number of positive and negative words endorsed and the recall of negative endorsed words were strong predictors of depressive symptoms in an adolescent, an undergraduate, and an adult sample. These results showed that SRP biases are robust predictors for depressive symptoms across age and sample types.

However, an important limitation in prior SRET research is the tendency to construe self-schemas and SRP biases as coherent, homogeneous variables by primarily investigating the relationship between the valence of one’s SRP biases and the underlying self-schemas and depression. While this approach is valuable in demonstrating a strong link between negatively-biased SRP and depression, it nonetheless ignores more nuanced individual differences in self-concepts. To our knowledge, studies using SRET have not investigated the specificity of one’s self-concepts beyond positive and negative valences in relation to depressive symptoms and subtypes of depression.

Self-concept has also been conceptualized as personality traits. The Five Factor Model of personality (FFM) is a widely recognized framework that categorizes personality traits into five broad dimensions: Extraversion, Agreeableness, Conscientiousness, Neuroticism, and Openness to Experience (McCrae and John, 1992). A meta-analysis revealed that depressed patients exhibited higher neuroticism, lower extraversion, lower conscientiousness, and no differences in agreeableness and openness as compared to non-depressed individuals (Kotov et al., 2010). Further, a study showed that neuroticism as indicated by the revised NEO Personality Inventory (NEO PI-R; Costa and McCrae, 2008) was positively correlated with depression scores measured by Beck’s revised Depression Inventory (BDI-II), whereas conscientiousness was negatively correlated with depressive severity among depressed individuals (Jourdy and Petot, 2017). Similarly, another study showed that among 10 community samples, extraversion and conscientiousness were negatively associated with depressive symptoms, while neuroticism was positively associated with depressive symptoms (Hakulinen et al., 2015). There is a high concordance between self-ratings of personality trait adjectives and self-reported personality questionnaires for the five personality dimensions (McCrae and John, 1992).

### Self-concepts and theoretical subtypes of depression

Research also indicates that distinct self-concepts may underlie theoretical subtypes of depression, such as Blatt and colleagues’ two personality subtypes of depression: dependent (or “anaclitic”) and self-critical (or “introjective”) depression (Blatt and Zuroff, 1992). While the dependent subtype focuses on interpersonal-relatedness and is characterized by fear of abandonment, longing for care from others, loneliness, and helplessness, the self-critical subtype is characterized by self-criticism, feelings of unworthiness and failure, and need for achievement and approval (Blatt and Zuroff, 1992; Abramson et al., 1997). Previous theoretical research suggested that depressed individuals with concerns in different dimensions (interpersonal vs. self-focused) are likely to use different self-referent adjectives to describe themselves (Dobson, 1986). Thus, it is worth investigating whether these two personality subtypes of depression can be distinguished through clustering the words that people endorse in SRET.

### The utility of a clustering approach to investigate subtypes of self-concepts in relation to depressive symptoms and depression subtypes

Given the central role that self-schemas play in depression, a more fine-grained analysis of the relationship between self-concepts, depressive symptoms, and depression subtypes through a clustering approach is both conceptually and clinically useful. First, by identifying natural subgroups based on SRET endorsement data in a clinical sample with elevated depressive symptoms, clustering allows us to explore if heightened depressive symptoms are underlined by an overall bias toward negative information and against positive information, or driven by specific patterns of self-concepts. Further, comparing the self-concepts of clusters of individuals in a clinical versus a non-clinical sample will provide further evidence of the kinds of self-concepts that underlie clinical symptoms of depression.

In terms of clinical utility, due to the high non-response and drop-out rates for psychotherapy among depressed patients (De Maat et al., 2007; Hans and Hiller, 2013; Cooper and Conklin, 2015), it is crucial to take into account individual idiosyncrasies to develop better personalized treatments for patients with depression. Investigating subtypes of self-concepts is especially useful for this purpose, since negative self-referential cognitions are an important target of cognitive-behavioral therapy (Yoshimura et al., 2014). Thus, a bottom-up approach looking at each individual’s data of unsupervised clustering may uncover patterns of self-concepts. Subsequently, if we can profile people based on their self-endorsement patterns and examine how their different self-concepts relate to depressive symptoms and depression subtypes, then we can better personalize treatment. Specifically, we can not only identify people who are more vulnerable to heightened depressive symptoms, but also identify specific aspects of their self-concepts to tackle, to improve treatment efficiency.

### The present study

The way in which biases in self-schemas and self-referential processing may contribute to the heterogeneity of depression is not well-understood. Since both self-concepts and depression are complex, heterogeneous constructs (Delugach et al., 1992; Bracken et al., 2000; Rush, 2007), the present study aims to contribute to the literature by using a clustering approach to examine the content of one’s self-concepts beyond valence, as indicated by self-endorsement of adjectives in SRET in relation to depressive symptoms. This may help to uncover subgroups of individuals with specific constellations of self-concepts who are more vulnerable to depression.

Additionally, we also investigate recall bias, which pertains to participants’ memory of self-referential adjectives. We aim to examine whether there are significant differences between the identified clusters in terms of their memory biases. However, our clustering approach aligns with prior research, which has highlighted the limitations of recall bias as a consistent metric, when compared to endorsement data. Notably, a study by Dainer-Best et al. (2018) found that the number of positive or negative words remembered was not strongly associated with the severity of depressive symptoms. Models solely based on recall bias data explained relatively little variance, compared to models looking at endorsement data. Consequently, the current study focuses on the pattern of endorsements as the basis for clustering individuals, rather than the pattern of memory biases.

Since self-concepts are multidimensional and heterogeneous (Delugach et al., 1992; Bracken et al., 2000) and depression tends to be associated with certain domains of self-concepts but not others (Beck, 1967, 1987), the richness of self-endorsement data in SRET may offer us more nuanced insights into the relationship between specific self-concepts and depression. Specifically, through a clustering approach, we can uncover whether there are naturally existing, data-driven subtypes of self-concepts that differentially relate to depression, and find the extent to which they agree with theorized subtypes of depression. Identifying subtypes of self-concepts that are more strongly related to depression and depression subtypes can then inform personalization of clinical treatments by tackling specific aspects of one’s self-concepts, and in turn, improve treatment efficacy.

Given the richness of self-referential data in SRET and the lack of studies investigating the relationship between clusters of self-concepts in relation to subtypes of depression, the present study aims to examine whether a clustering approach can reveal meaningful subtypes of self-concepts that differentially relate to depression severity and subtypes of depression, using three existing Singapore-based datasets that are later reconfigured into a clinical and a non-clinical group. Clusters are first generated using the self-endorsement data in SRET based on the clinical, non-clinical, and overall samples, and reliability and correspondence of the clusters are examined. Subsequently, the characteristics of each cluster are examined to see if meaningful subtypes of self-concepts were revealed through the clusters. The clusters are also compared on their level of positive/negative endorsement and other clinical measures to investigate what the clusters are associated with.

We expect that clinical and non-clinical clusters will show good stability and correspondence when compared to combined clusters. Further, different patterns of self-endorsement are expected to be revealed between clinical and non-clinical clusters. We also hypothesize that within clinical and non-clinical clusters, clusters will show differences in their endorsement of positive and negative words, depressive symptoms, as well as the two personality subtypes of depression.

## Methods

### Participants

Anonymized data was pooled from four studies at IMH and NUS where questionnaire and task-based data were collected under similar conditions. Both IMH studies, Study Reference Number: 2015/00545, 2018/01184 and 2021/00005, were approved by the IMH Institutional Research Review Committee (IRRC) and NHG Domain Specific Review Board (DSRB) and informed written consent was obtained from participants. The NUS sample was collected with approval from the NUS Institutional Review Board and Psychology Department Ethics Review Committee (DERC) under Psych-DERC Reference Code: 2018-August-86.

The clinical sample comprised 119 patients from the Institute of Mental Health (IMH) with past or current anxiety or depressive symptoms who were literate in English. They were recruited from triage, outpatient clinics, and referrals from therapists. They were recruited from three studies: 85 IMH patients from the “Understanding the person, exploring change across psychotherapies” (Xchange) study, which included data from the “Understanding the Person, Improving Psychotherapy: Preventing Relapse by targetting Emotional bias Modulation in PsychoTherapy” (PRE-EMPT) and 34 patients and 18 healthy controls from “The role of cholinergic dysfunction in the progression of depression” (CholDep) study. In the Choldep study, healthy controls were also recruited by word of mouth.

The non-clinical community/university sample comprised 97 participants mainly recruited at the National University of Singapore as part of an undergraduate thesis project. For the purposes of clustering analysis, this sample was merged with the 18 healthy controls from the CholDep project to form a total of 115 participants in the ‘non-clinical sample’. A meta-analysis reported that the relationship between implicit cognitive biases and depression showed similar effect sizes in studies with clinical, community, and undergraduate samples (Phillips et al., 2010). The purpose of a non-clinical sample was to identify how clustering solutions differed across disparate clinical and non-clinical populations.

### Procedure

Participants in all samples completed questionnaires and the Self-Referential Encoding Task (SRET) remotely online using the Inquisit platform (Inquisit 5 [Computer software], 2016) by Millisecond. The procedure consisted of the following steps. First, during the endorsement phase of the SRET, participants were presented with one word at a time in a random order and indicated whether the word described them by pressing a corresponding keyboard button. Following the endorsement phase, participants worked on a digit-symbol substitution distractor task for 5 min. After the distractor task, participants were asked to recall as many words as possible from the endorsement phase.

### Measures

#### Self-referential encoding task (SRET)

The SRET is a computer-based task used to access one’s self-relevant schemas (Derry and Kuiper, 1981) that typically includes three segments in order: endorsement, distractor task, and incidental recall. In a given trial of the endorsement phase, participants judged whether presented adjectives described them (“Describes me?”). The SRET presented both positive (e.g., “popular,” “successful”) and negative adjectives (“awful,” “ugly”) in random order. The participants responded by pressing “Yes” or “No” keys on a computer keyboard. Participants’ responses and reaction time (measured in ms) were recorded for each trial. After the endorsement phase, participants worked on a distractor task for 5 min to minimize interference and memory consolidation of the endorsed words before undertaking the incidental recall task.

Three SRET metrics were calculated to assess the responses in the SRET, namely the endorsement rate, reaction time (RT) and recall bias.

Endorsement rate, represents the proportion of positive/ negative words that participants endorsed as describing themselves. It was calculated as the number of positive/ negative words, divided by the total number of words presented to the participants.

Two RT variables, Negative RT bias and Positive RT bias were calculated to assess participants’ reaction time differences between endorsing and rejecting negative/ positive words during the SRET. The formula for Negative RT bias is as follows: Negative RT Bias = (Mean RT of Endorsement of Negative Words − Mean RT of Rejection of Negative Words) / Average RT Across all Trial Types. Similarly, Positive RT Bias was calculated using the following formula: Positive RT Bias = (Mean RT of Endorsement of Positive Words − Mean RT of Rejection of Positive Words) / Average RT Across all Trial Types. This method of calculating RT bias aligns with the approach used in prior SRET studies (Connolly et al., 2016).

Recall bias was computed by dividing the total number of either positive or negative words endorsed and recalled by the total number of words endorsed and recalled. Only words that were correctly recalled were considered. Positive recall bias was obtained by dividing the number of positive recalled words by the total recalled words, while negative recall bias was calculated by dividing the number of negative recalled words by the total recalled words. A difference between negative recall and positive recall bias was also taken to measure the relative strength of memory biases for positive and negative self-referential information.

While the standard SRET was administered in the XChange and Choldep studies, some variations to the SRET were used in the University sample. Besides the typical three segments, the SRET in the XChange and Choldep also included an additional endorsement task in a matrix format where participants were presented with a matrix of words at once and were asked to tick the box under words that they identified themselves with. 60 words were presented in the first endorsement task, and 200 words were presented in the matrix task. The 200 words consist of 88 personality-trait words (Anderson, 1968), 24 trait-adjectives (Frewen et al., 2011), 11 from SRET words validated in predicting depressive relapse (LeMoult et al., 2017), 16 adjectives from the Revised Interpersonal Adjective Scales: Big Five Version (IASR-B5; Trapnell and Wiggins, 1990), and the remainder derived from the List of Threatening Experiences (LTE, Brugha and Cragg, 1990) and the Wheel of Emotions (Plutchik, 2001).

The SRET in the undergraduate-student sample included 179 words in the endorsement task, 40 words from LeMoult et al. (2017), 120 words from IASR-B5 (Revised), and 19 words from the List of Threatening Experiences (LTE, Brugha and Cragg, 1990). The processing of SRET data into a standardized and comparable format is explained in a later section on data processing.

### Depressive symptoms

IDS-30-SR is a 30-item self-report measure of depressive symptoms that includes all criterion symptoms for a major depressive episode, as well as all criterion symptoms for melancholic and atypical subtypes of depression (Rush et al., 1996). Participants were asked to rate the severity of each of the 30 symptoms in the preceding 7 days on a scale of 0–3, with higher scores indicating greater symptom severity. The total score is calculated by summing 28 of the 30 items (for the appetite and weight change questions, only appetite and weight increase *or* decrease was scored for any participant). The total score ranges from 0 to 84. IDS-30-SR was shown to have satisfactory psychometric properties: Cronbach’s alpha was 0.94 for an overall sample including depressed individuals and healthy controls, and 0.77 for symptomatic-only individuals, indicating acceptable internal consistency across depressed and non-depressed individuals (Rush et al., 1996). IDS-30-SR also highly correlates with other self-reported scales measuring depressive symptoms such as 17-item HRS-D (*r* = 0.88, *p* < 0.0001) and BDI (*r* = 0.93, *p* < 0.0001), showing good convergent validity (Rush et al., 1996). IDS-30-SR was also demonstrated to significantly discriminate between symptomatic depressed individuals and non-symptomatic euthymic individuals. The suggested optimal cut-off score for IDS-30-SR is 18, as determined by ROC analysis, with a sensitivity of 1.0 and specificity of 0.94 (Rush et al., 1996). People scoring 18 and above are considered symptomatically depressed. The numbers of people who met this cut-off score in the clinical and non-clinical groups are reported in this study. In the current study, Cronbach’s alpha for IDS-30-SR is 0.909 for the clinical group, 0.912 for the non-clinical group, and 0.938 for the overall sample.

### Depression subtypes

The Reconstructed Depressive Experiences Questionnaire (RecDEQ) is a 19-item measure that differentiates between the dependent (anaclitic) and self-critical (introjective) personality subtypes of depression (9 items for dependency, 10 items for self-criticism). Sample items for the dependency scale include: “I become frightened when I feel alone,” and sample items for the self-criticism scale include: “I tend not to be satisfied with what I have.” Participants were asked to rate each item on a Likert scale of 1–7 (1 = strongly disagree, 7 = strongly agree). All items for each of the two scales are summed to obtain a total score for each scale. RecDEQ showed excellent fit to a two-factor model in both an undergraduate and a clinical sample, and an association with depressive symptoms that are in line with theoretical predictions (Desmet et al., 2007). Test–retest reliability was 0.75 for the dependency scale, and 0.83 for the self-criticism scale. Cronbach’s alpha for the two scales ranged from 0.69 to 0.80 across four samples (normal adults, university students, depressed patients, and panic disorder patients; Bagby et al., 1994).

### Data processing and reorganization

To maximize the self-endorsement data used as input in the clustering analysis, endorsement data in the matrix task in XChange and Choldep were included to identify the maximum overlap across three samples. Endorsement data on 90 overlapping words (48 negative and 42 positive) across all participants were used in the clustering analyses (see the word list in Supplementary Appendix 1).

For grouping of participants, all participants in XChange and the clinical participants in Choldep were combined to form the clinical group, and all participants in the undergraduate sample and healthy control participants in Choldep were combined to form the non-clinical group.

Demographic variables, SRET variables, namely endorsement rate, reaction time and recall bias (participants’ ability to recall positive or negative self-referential adjectives), and depressive symptom severity were examined and compared to confirm that participants grouped into the same group were similar on these measures and that the grouping decision was reasonable. See Supplementary Table S1 for the full list of demographic and clinical characteristics of each subsample and Supplementary Table S2 for correlations of age and SRET variables with depressive symptoms.

### Clustering analysis methodology

The clustering analysis was conducted separately for the clinical-only sample, the non-clinical-only sample and the combined sample with the Rmixmod package in R Studio. Rmixmod is a package devoted to clustering (or, unsupervised classification) using mixture modeling. Other approaches for clustering including K-means clustering, hierarchical clustering and Gaussian models were considered for clustering analysis but given that we had 10 multivariate multinomial mixture models, Rmixmod was deemed the most suitable method as it could effectively manage high-dimensional binary data. Rmixmod specializes in finite mixture modeling and latent class analysis, making it well-suited for data that arises from multiple underlying distributions, like in the case of our binary SRET endorsement data (Lebret et al., 2015). Under Rmixmod package, mixmodCluster() function was used to obtain clustering solutions. Arguments required for the function included the criterions used for optimization of models and seed number that specifies generation of a particular sequence of numbers.

Optimization of models was done according to Bayesian Information Criterion (BIC), Integrated Completed Likelihood (ICL), and Normalized Entropy Criterion (NEC). BIC, a widely-used statistical criterion used for model selection, aims to strike a balance between model fit and model complexity (Neath and Cavanaugh, 2012). Lower BIC values indicate a better fit to the data while remaining parsimonious, making models with the lowest BIC value preferable. ICL, another model selection criterion, is typically used in mixture modeling and clustering analyses (Biernacki et al., 2000). It evaluates the quality of clusters with higher ICL values suggesting more distinct and better-defined clusters, indicating a more appropriate clustering solution. NEC, on the other hand, is a model selection criterion that measures the quality of clusters by assessing the dispersion of data points within them (Biernacki et al., 1999). Lower NEC values indicate more compact and well-separated clusters, which are considered better for clustering solutions.

Clustering solutions were generated for Seed 1–30, and each set of solutions were sorted according to the 3 criterions used for optimisation of models. To compare the solutions, criterion values were extracted. We also considered the likelihood values of each solution as a measure of how well the clustering model explains the observed data, thus a higher likelihood value indicates better clustering. Two clustering evaluation metrics were used to determine the optimisation model. It includes the Davies-Bouldin index (DBI) and Calinski-Harabasz Index (CHI). The Davies-Bouldin index (DBI) is calculated as the average similarity of each cluster with its most similar cluster. A lower DBI value means the clusters are better separated (Davies and Bouldin, 1979). The Calinski-Harabasz Index (CHI) is a variance ratio criterion that evaluates the ratio of between-cluster variance and within-cluster variance. A higher value suggests a better clustering solution (Caliński and Harabasz, 1974).

Following Bozdogan’s (1993) recommendation, the number of clusters tested in each clustering analysis ranged from 2 to the smallest integer larger than the cube root of the number of observations in each sample. Based on this guideline, the number of clusters tested in both the clinical and non-clinical sample was between 2 and 5, and the number of clusters tested in the combined sample was between 2 and 7. The lowest criterion value was used to choose the optimal number of clusters.

Clustering analyses were run on the clinical, non-clinical, and overall sample, respectively, to identify meaningful subgroups of participants based on their self-endorsement patterns in SRET.

### Analyses

A simplified thematic analysis using a deductive approach was conducted on the top-endorsed words by each clinical and non-clinical cluster to characterize the patterns of self-concepts in each cluster (Boyatzis, 1998; Braun and Clarke, 2006) by mapping the most endorsed words in each cluster to the five-factor model of personality (McCrae and John, 1992) and Blatt’s two personality subtypes of depression (Blatt and Zuroff, 1992). Words with a mean endorsement equal to or above the upper quartile of mean endorsements of words within a cluster were defined to be the top-endorsed words for a cluster. These top-endorsed words were then reviewed and mapped onto the five dimensions of the Five Factor Model of Personality (FFM) and the two personality subtypes of depression (dependent and self-critical).

Correspondence of the clusters were examined by comparing the clinical and non-clinical clustering solutions to the overall sample clusters.

One-way univariate between-subject ANOVAs on positive and negative endorsement and depressive symptoms were conducted on the clinical and non-clinical clusters, respectively. Post-hoc tests, such as Tukey’s Honestly Significant Difference (HSD), were also conducted for pairwise comparisons following the ANOVAs. This test corrects for the inflation of Type I error inflation that can occur when conducting multiple pairwise comparisons. It applies a rigorous correction method that considers the overall error rate and ensures that the observed differences between group means are truly statistically significant. To examine for the differences in the frequency of the different diagnoses between the clusters, a multinomial test was conducted on the clinical clusters, using JASP 0.16.3.

## Results

### Demographics and clinical characteristics of participants

The demographic and clinical characteristics of participants in the clinical and non-clinical group are presented in Table 1. The two groups significantly differed in age and gender distribution, where the clinical group was significantly older than the non-clinical group (*t*(144) = −7.68, 95%CI [−10.44, −6.16], *p* < 0.0001, *d* = −0.99) and the non-clinical group had significantly more female participants (*χ*^2^ = 5.87, df = 1, *p* = 0.015). The effect of age and gender was therefore checked and controlled in subsequent analyses. As expected, the non-clinical group (M = 14.56, SD = 11.59) had significantly lower depressive symptom scores than the clinical group (M = 31.25, SD = 13.90; *t*(232) = −9.96, 95%CI [−20.00, −13.39], *p* < 0.0001, *d* = −1.30). There was no significant gender by group interaction (*t*(232) = 0.48, 95%CI [−3.00, 4.98], *p* = 0.62).

**Table 1:** Demographic and clinical characteristics.

	Clinical sample (*N* = 119)		Non-clinical sample (*N* = 115)	
	*n*	%	*n*	%
Demographic characteristics				
Sex				
Male	60	50.4	39	33.9
Female	59	49.5	76	66.1
Ethnicity				
Chinese	95	80	101	87.8
Malay	8	6.7	3	2.6
Indian	8	6.7	5	4.4
Others	8	6.7	6	5.2

	Mean	SD	Mean	SD
Age	32.38	11.17	24.08	3.70
Clinical characteristics				
Depressive symptoms, IDS-30-SR	31.25	13.90	14.56	11.59

	*n*	%	*n*	%
Diagnoses in clinical sample				
Anxiety (without depression)	14	11.8		
Depression (without anxiety)	62	52.1		
Mixed anxiety and depression	9	7.6		
Adjustment disorder	21	17.6		
Bipolar disorder	7	5.9		
Obsessive compulsive disorder (OCD, OCD with depression, OCD with Tourette’s, OCD with anxiety and depression)	6	5.0		
Additional comorbid psychiatric diagnoses (schizophrenia, cluster B traits, eating disorder, obsessive-compulsive personality disorder, gambling, alcohol dependence, substance abuse, insomnia, attention deficit disorder with hyperactivity)	10	8.4		

M, mean; SD, Standard Deviation. % Percentage of each category out of total. Clinical diagnoses are categorized into five groups (depression, anxiety, mixed anxiety and depression, adjustment disorder and bipolar disorder). Additional comorbid psychiatric diagnoses (schizophrenia, cluster B traits, eating disorder, obsessive-compulsive personality disorder, gambling, alcohol dependence, substance abuse, insomnia, attention deficit disorder with hyperactivity) were accounted for.

### Clustering evaluation and stability

The cluster validation metrics for goodness of split and stability are presented in Table 2. When we performed perturbation by varying the seed for initialisation across 30 seeds, we identified that optimisation with BIC and ICL yielded the most stable solutions with no differences in the number of clusters identified and minimal differences in criterion values. The optimum number of clusters differed between seeds when using NEC and variance across seeds was relatively higher. While BIC and ICL yielded similar DBI and CHI values, the BIC criterion values and likelihood values showed less variance between seeds. Thus, BIC optimization was used for the final clustering solution. We also examined the centroid locations and in the majority of the seed to seed comparisons, a unique centroid location with correlation coefficient of 0.7 or above was identifiable for a given cluster. The clustering solution for seed number 3 was chosen as a representative solution as the various solutions appeared to have trade-offs between metrics.

**Table 2:** Descriptive statistics of cluster validation metrics for goodness of split and stability.

	Number of clusters (Mean, S.D.)	Criterion value (Mean, S.D.)	Likelihood (Mean, S.D.)	DBI (Mean, S.D.)	CHI (Mean, S.D.)
Clinical					
BIC	5,0	11659.93, 14.29	−5621.75, 56.13	0.049, 0.0078	6.44, 0.21
ICL	5,0	11682.76, 17.65	−5628.60, 67.44	0.049, 0.0078	6.44, 0.21
NEC	4.73, 0.45	0.03, 0.01	−5179.5, 47.98	0.054, 0.0072	7.26, 0.49
Non-clinical					
BIC	5,0	11154.12, 13.07	−5562.98, 8.05	0.045, 0.013	7.37, 0.17
ICL	5,0	11179.56, 14.90	−5562.7, 8.31	0.045, 0.013	7.39, 0.17
NEC	4.93, 0.25	0.018, 0.0032	−4932.34, 23.56	0.078, 0.0044	7.91, 0.39
Combined					
BIC	7,0	23123.03, 47.02	−11515.96, 70.35	0.046, 0.011	13.40, 0.25
ICL	7,0	23183.17, 49.44	−11514.91, 69.47	0.045, 0.011	13.40, 0.25
NEC	6.53, 0.63	0.016, 0.0027	−10365.17, 89.22	0.062, 0.0048	14.66, 1.14

Mean and standard deviation of the cluster validation metrics for clustering solution of seed 1–30.

Broadly, the majority of algorithms generated 5 clusters as the optimal solution for both the clinical (C1–C5) and non-clinical group (N1–N5). 7 clusters were generated as the optimal solution for the combined sample (NC1–NC7). All three optimal clustering solutions have the maximum possible number of clusters for their respective sample size.

### Top endorsed words in clinical, non-clinical and combined clusters

In order to characterize and label the clusters, we ranked words by both mean endorsement rates and relative endorsement rates between clusters (See Supplementary Appendix 2). The most representative words have been listed in Table 3.

**Table 3:** Top endorsed words for every cluster.

Clinical						
C1 Neurotic (*N* = 17)	C2 Extraverted (*N* = 24)	C3 Anxious to please (*N* = 13)	C4 Self-critical (*N* = 49)	C5 Conscientious (*N* = 16)		
Cruel Fretful Systematic Unsparkling Uncheery Impractical High-strung	Enthusiastic Successful Unnervous Lively Exciting Winner Happy	Cheerful Timid Shy Worrying Nice Introverted Nervous	Awful Coward Inefficient Abandoned Stupid Bad Ugly	Relaxed Secure Successful Unworrying Self-confident At ease Calm		
Non-clinical						
N1 (Self-confident) (*N* = 23)	N2 (Low endorsement) (*N* = 21)	N3 (Non-neurotic) (*N* = 27)	N4 (Neurotic) (*N* = 21)	N5 (High endorsement) (*N* = 23)		
Popular Successful Unworrying Attractive Well-Off Leader Winner	Abandoned Boring Inefficient Anxious Quiet Awful Hurt	Unworrying At Ease Relaxed Unnervous Self-Assured Neighbourly Lucky	Ugly Unsparkling Coward Impractical Loss Meek Upset	Bad Ill Accused Cruel Unkind Hurt Angry		
Combined						
NC1 (Self-confident) (*N* = 38)	NC2 (Externalising) (*N* = 23)	NC3 (Neurotic) (*N* = 20)	NC4 (Secure) (*N* = 46)	NC5 (Low endorsement) (*N* = 42)	NC6 (High endorsement) (*N* = 13)	NC7 (Self-critical) (*N* = 52)
Unworrying Successful Winner At Ease Popular Unnervous Stable	High-Strung Leader Impractical Angry Cruel Burdened Exciting	Impractical Unsparkling Uncharitable Ill Uncheery Victim Fretful	Secure Unworrying Unnervous Relaxed At Ease Neighbourly Stable	Unkind Coward Boring Hurt Inquisitive Quiet Smart	Victim Tricky Cruel Uncheery Fretful Burdened Loss	Awful Abandoned Bad Useless Stupid Ugly Coward

Five clinical clusters, five non-clinical clusters and seven combined clusters were generated from clustering analysis. Clusters are labeled according to the top endorsed words. Top-endorsed words have a mean relative endorsement rate equal to or above the upper quartile of mean relative endorsements of words within a cluster.

The clinical clusters were labeled as “Neurotic” (C1), “Extraverted” (C2), “Anxious to please” (C3), “Self-critical” (C4), “Conscientious” (C5). In C1, words associated with neuroticism such as “fretful,” “uncheery” and “high-strung” were highly endorsed as were the number of self-critical words. C2 individuals endorsed more positive words related to extraversion, such as “enthusiastic,” “unnervous” and “lively.” C3 individuals were labeled as “anxious to please” because they endorsed words related to agreeableness, such as “cheerful” and “nice”; introversion such as “introverted,” “timid” and “shy”; and anxiety such as “nervous” and “worrying.” C4 members endorsed negative self-critical words like “awful,” “stupid,” “bad” and “ugly.” Individuals in C5 endorsed more words associated with conscientiousness such as “secure,” “successful” and “self-confident.”

The non-clinical clusters were labeled as “Self-confident” (N1), “Low endorsement” (N2), “Non-neurotic” (N3), “Neurotic” (N4), “High endorsement” (N5). In N1, members highly endorsed positive words like “popular,” “successful,” “attractive” reflecting self-assuredness and a positive self-image. In N2, negative words like “abandoned,” “boring,” “inefficient” were highly endorsed, and overall had a low endorsement rate (36.6%). In N3, words like “Unworrying,” “Relaxed,” “Self-Assured,” and “Neighbourly” indicate a lack of anxiety, stress, and emotional turbulence, which are characteristic of non-neurotic individuals. Conversely, individuals in N4 endorsed more neurotic words such as “ugly,” “unsparkling,” “impractical” which collectively convey a sense of negativity and emotional distress that are often associated with neurotic tendencies. Lastly, individuals in N5 had the highest mean endorsement rate (58.1%) and they endorsed both negative and positive words at a higher rate compared to the other non-clinical clusters.

The combined clusters were labeled as “Self-confident” (NC1), “Externalising” (NC2), “Neurotic” (NC3), “Secure” (NC4), “Low endorsement” (NC5), “High endorsement” (NC6), “Self-critical” (NC7). NC1 individuals highly endorsed positive words like “unworrying,” “successful,” “winner,” reflecting their high self-confidence. Individuals in NC2 endorsed words reflecting externalizing behavior such as “high-strung,” “leader,” “impractical,” “angry.” In NC3, words associated with neurotic tendencies such as “ill,” “fretful” and “uncharitable” were highly endorsed. Conversely, individuals in NC4 highly endorsed words like “secure,” “unworrying,” “unnervous,” “relaxed,” indicating a sense of security and emotional stability. Among the clusters, individuals in NC5 exhibited the lowest endorsement rate (32.1%), while individuals in NC6 demonstrated the highest endorsement rate (67.7%). Lastly, individuals in NC7 highly endorsed self-critical words like “awful,” “bad,” “useless,” “stupid,” “ugly.” (See Supplementary Appendix 3 for mapping of top-endorsed words to personality).

A closer examination at the top words endorsed by the combined sample revealed significant overlaps with the clinical and non-clinical clusters. NC1, the “Self-confident” cluster endorsed the same words as N1 (Self-confident): “unworrying,” “successful,” “popular” as well as N3 (Non-neurotic): “at ease.” Similarly, NC4, the “Secure” cluster also endorsed the same words as N3: “relaxed,” “at ease.” In the case of NC7, the “Self-critical” cluster, there were overlaps with C4 (Self-critical): “awful,” “coward,” “abandoned,” “stupid,” “bad,” “ugly.” Given that there is a significant overlap in the top words endorsed, we did a correspondence analysis between the clustering solutions.

### Correspondence between clinical, non-clinical and combined clustering solutions

The mapping of clinical (C) and non-clinical clusters (NC) with the clusters in the combined sample (NC) revealed that there are both important overlaps and differences between the clinical and non-clinical clusters. 95% of NC1 (Self-confident) (*N* = 38) are made up of participants from N1 (Self-confident) (*N* = 23); N3 (Non-neurotic) (*N* = 11), while 89% of NC4 (Secure) (*N* = 46) are made up of participants from N2 (Low endorsement) (*N* = 12), N3 (Non-neurotic) (*N* = 15), N4 (Neurotic) (*N* = 6), and N5 (High endorsement) (*N* = 8). Specifically, NC1 (Self-confident) and NC4 (Secure) are primarily composed of non-clinical participants, reflecting traits like high extraversion and low neuroticism. In contrast, NC5 (Low endorsement) and NC7 (Self-critical) consisted mainly of clinical participants, showing higher endorsement in dependency and self-criticism traits. 74% of NC5 (Low endorsement) (*N* = 42) are made up of participants from C2 (Extraverted) (*N* = 8), C3 (Anxious to please) (*N* = 6), C4 (Self-critical) (*N* = 6), and C5 (Conscientious) (*N* = 11); and 96% of NC7 (Self-critical) (*N* = 52) are made up of participants from C3 (Anxious to please) (*N* = 6) and C4 (Self-critical) (*N* = 43). Meanwhile, other combined clusters are represented by a roughly equal mix of clinical and non-clinical participants, including NC2 (Externalising) (65% clinical), NC3 (Neurotic) (50% clinical), and NC6 (High endorsement) (46% clinical).

In the combined (NC) sample solution, two clusters fully included clusters from the clinical (C) or non-clinical (N) solutions: NC1 (Self-confident)/ N1 (Self-confident), NC7 (Self-critical)/ C4 (Self-critical), and an additional three were dominated by two clusters (NC2 (Externalising)/ C2 (Extraverted)/ N5 (High endorsement), NC3 (Neurotic)/ C1 (Neurotic)/ N4 (Neurotic), NC6 (High endorsement)/ C1 (Neurotic)/ N5 (High endorsement)), and two were comprised of several clusters. While the combined solution had two clusters comprising primarily the non-clinical sample (NC1 (Self-confident)/ N1 (Self-confident), NC4 (Secure)/ N2 (Low endorsement)/ N3 (Non-neurotic)/ N5 (High endorsement)) and one comprising a clinical cluster (NC7 (Self-critical)/ C4 (Self-critical)), there was meaningful co-occurrence of clinical with nonclinical cluster in the combined solution as well (C1 (Neurotic)/ N4 (Neurotic), C2 (Extraverted)/ N5 (High endorsement), C3 (Anxious to please)/ C5 (Conscientious)/ N2 (Low endorsement)).

**Figure 1:**
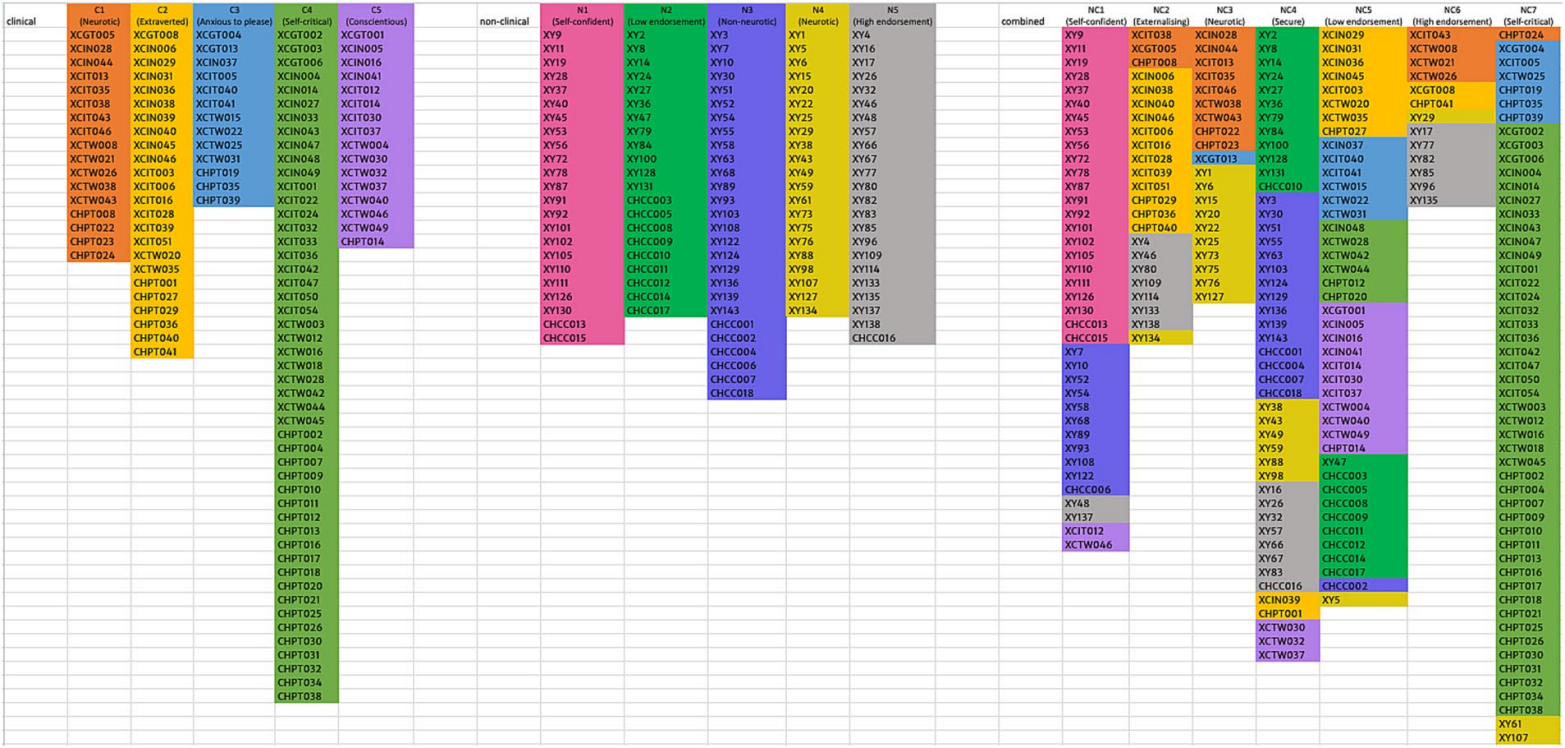
Mapping of clinical and non-clinical clusters onto combined clusters.

Furthermore, the distribution of clusters within the combined solution demonstrated varying levels of preservation. For example, C2, C3, and C4 are each primarily distributed only in two combined clusters and are thus well-preserved. Contrarily, C1 is distributed across four combined clusters (NC2, NC3, NC6, NC7), and C5 is distributed across three combined clusters (NC1, NC4, NC5); thus, these two clinical clusters are less well-preserved in the combined solution. For non-clinical clusters, N1 is only found in NC1, while N2 and N3 are each found primarily in only two combined clusters, thus relatively well-preserved. In contrast, N4 and N5 are distributed across six and four combined clusters respectively, less well-preserved. However, even for the less well-preserved clusters, they tend to be predominantly distributed across only two to three combined clusters, which suggests a certain degree of cluster consistency for these clinical and non-clinical clusters. Refer to Figure 1 for the mapping of clinical and non-clinical clusters onto combined clusters and Supplementary Figure S1 for reverse mapping.

### Cross-cluster differences in negative and positive endorsement

Cluster differences in negative and positive endorsement were examined through scatter plots and one-way ANOVAs.

#### Clinical clusters

**Figure 2:**
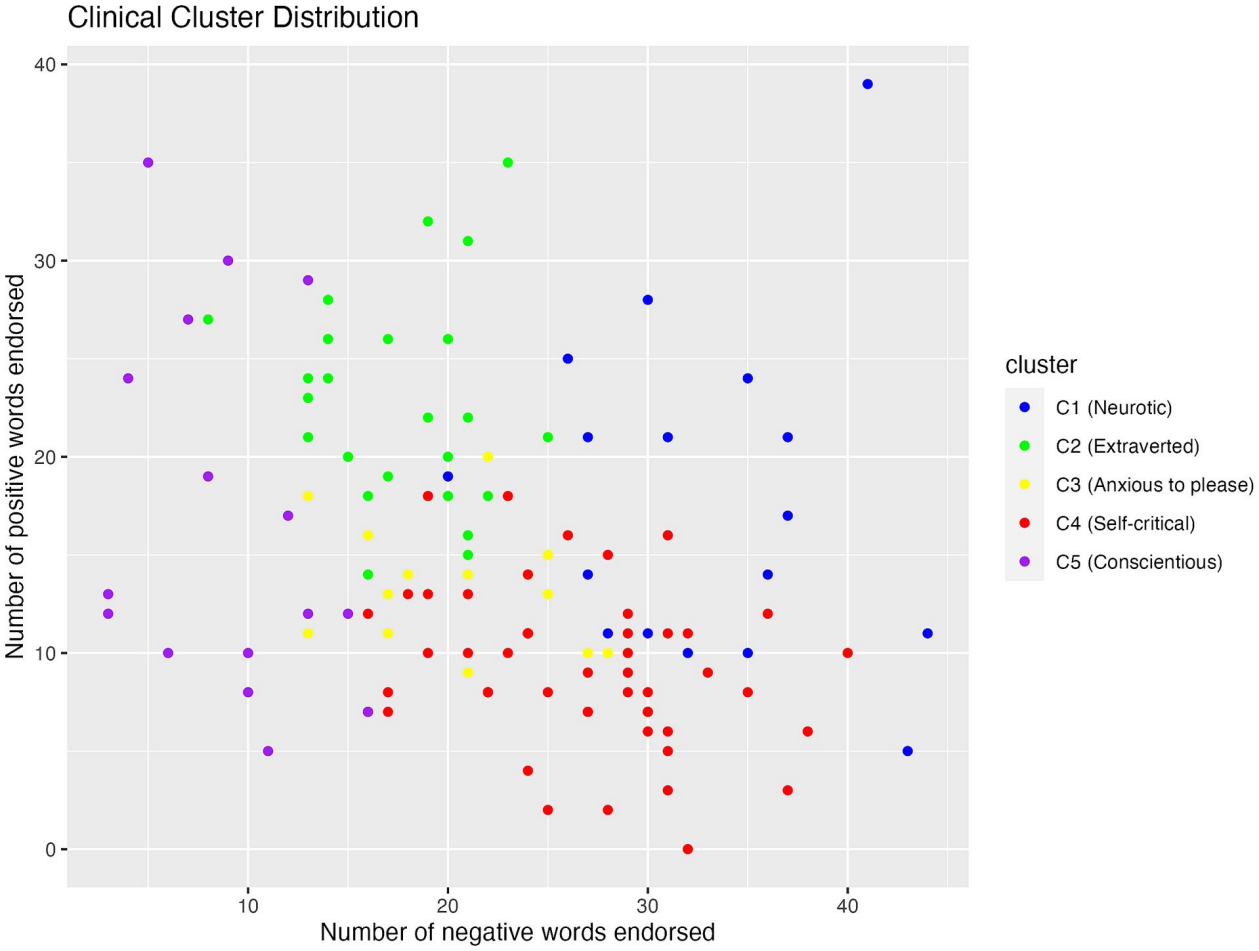
Scatterplot showing the distribution of positive and negative words endorsed by participants in the five clinical clusters.

The scatterplot of cluster distribution on the number of positive and negative words endorsed (see Figure 2) showed that C1 and C4 endorsed the most amount of negative words, followed by C2 and C3. C5 endorsed the least amount of negative words, but showed a wide spread of the number of positive words endorsed within this cluster.

One-way between-subject ANOVA revealed that clinical clusters were significantly different in the number of negative words they endorsed (*F* (4, 114) = 54, *p* < 0.001, *η*^2^ = 0.65; see Table 3). Post-hoc analysis using Tukey HSD revealed that among the clinical clusters, people in C1 (M = 32.88, SD = 6.48) endorsed significantly more negative words than all the other clusters (*p* < 0.001). C4 (M = 26.71, SD = 5.91) endorsed more negative words than C2 (M = 17.58, SD = 4.05) and C3 (M = 20.23, SD = 5.04, *p* < 0.01), both of which endorsed more negative words than C5 (M = 9.06, SD = 4.16, *p* < 0.001).

Clinical clusters also differed in the number of positive words they endorsed (F (4, 114) = 23, *p* < 0.001, *η*^2^ = 0.45; see Table 4). Specifically, C4 (M = 9.18, SD = 4.01) endorsed fewer positive words than C1 (M = 17.71, SD = 8.40), C2 (M = 22.75, SD = 5.40), and C5 (M = 16.88, SD = 9.32, *p* < 0.001), and C2 endorsed more positive words than C3 (M = 13.39, SD = 3.28), C4, and C5 (*p* < 0.05).

**Table 4:** Analysis of variance (ANOVA) results for the effect of clusters on endorsement rate in all samples.

Source	Sum of squares	*df*	Mean square	*F*	*η* ^2^	*p*
**Clinical**						
Number of negative words endorsed						
Cluster	6235.38	4	1558.84	54.00	0.66	<0.001***
Residuals	3290.84	114	28.87			
Number of positive words endorsed						
Cluster	3297.53	4	824.38	23.46	0.45	<0.001***
Residuals	4006.20	114	35.14			
**Non-clinical**						
Number of negative words endorsed						
Cluster	7832.20	4	1958.05	70.67	0.72	<0.001***
Residuals	3047.60	110	27.72			
Number of positive words endorsed						
Cluster	4837.18	4	1209.29	52.012	0.65	<0.001***
Residuals	2557.55	110	23.25			
**Combined**						
Number of negative words endorsed						
Cluster	16834.62	6	2805.77	97.93	0.72	<0.001***
Residuals	6503.72	227	28.65			
Number of positive words endorsed						
Cluster	18723.57	6	3120.60	168.80	0.82	<0.001***
Residuals	4196.50	227	18.49			

***p < 0.001.

#### Non-clinical clusters

**Figure 3:**
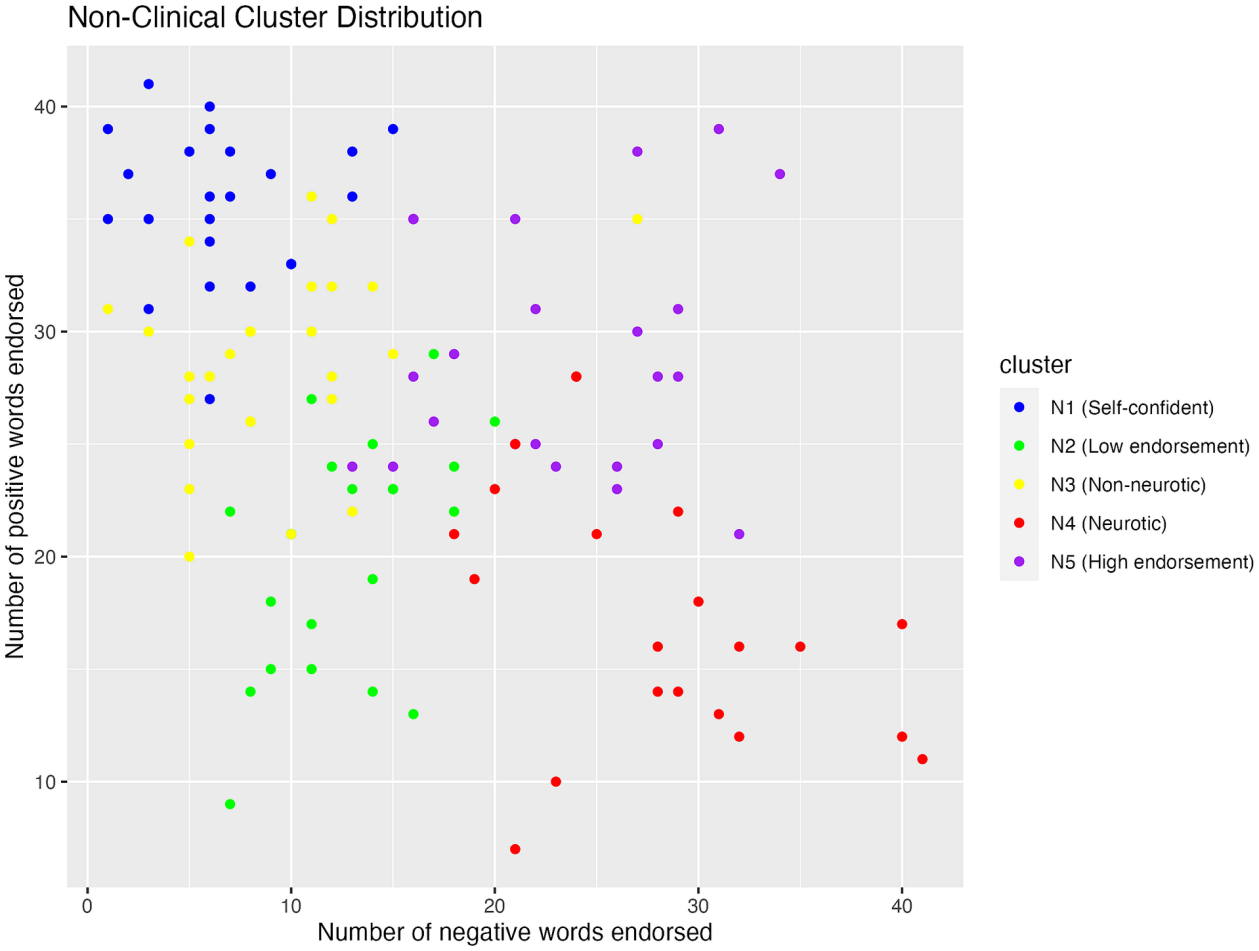
Scatterplot showing the distribution of positive and negative words endorsed by participants in the five non-clinical clusters.

The scatterplot of non-clinical cluster distribution (see Figure 3) showed that both N4 and N5 endorsed a high number of negative words, but N5 endorsed more positive words than N4. While N1, N2, and N3 fell in the same range for endorsing negative words, N1 endorsed the greatest number of positive words, followed by N3 and N2.

One-way between-subject ANOVA revealed that non-clinical clusters differed significantly in negative endorsement (*F* (4, 110) = 71, *p* < 0.001, *η*^2^ = 0.72; see Table 4). Specifically, N4 (M = 28.10, SD = 6.94) endorsed significantly more negative words than N5 (M = 23.35, SD = 6.10, *p* = 0.028), which in turn endorsed more negative words than N1 (M = 6.61, SD = 3.76), N2 (M = 12.81, SD = 3.78), and N3 (M = 9.07, SD = 5.08, *p* < 0.001). Further, N2 endorsed more negative words than N1 (M = 6.61, SD = 3.76, *p* = 0.0015).

Non-clinical clusters also differed in positive endorsement (F (4, 110) = 52, *p* < 0.001, *η*^2^ = 0.65; see Table 4). Specifically, N1 (M = 35.70, SD = 3.32) endorsed more positive words than all the other clusters (*p* < 0.001). Additionally, N3 (M = 28.667, SD = 4.18) and N5 (M = 28.91, SD = 5.32) endorsed more positive words than N2 (M = 20.14, SD = 5.31) and N4 (M = 17.29, SD = 5.81, *p* < 0.0001).

#### Combined clusters

One-way between-subject ANOVA was also conducted to see if the combined clusters differed significantly in endorsement rates. The results showed that combined clusters also differed significantly in negative endorsement (*F* (6, 227) = 97.93, *η*^2^ = 0.72; see Table 4). Specifically, NC3 (M = 34.00, SD = 5.32) endorsed more negative words compared to the other clusters (*p* < 0.001). NC7 (M = 27.17, SD = 5.30) also endorsed more negative words than NC1 (M = 7.11, SD = 4.39), NC2 (M = 20.26. SD = 5.55), NC4 (M = 14.61, SD = 6.16), NC5 (M = 14.24, SD = 5.11) and NC6 (M = 30.15, SD = 5.52, *p* < 0.001). On the flip side, NC1 endorsed fewer negative words compared to the other clusters (*p* < 0.001). NC4 endorsed fewer negative words than NC2, NC3, NC6 and NC7, while NC5 (M = 14.24, SD = 5.11) endorsed fewer negative words than NC2, NC3, NC4, NC6 and NC7.

Combined clusters also differed in positive endorsement (F (6, 227) = 168.80, *η*^2^ = 0.82; see Table 4). Specifically, NC1 (M = 33.76, SD = 4.43) endorsed significantly more positive words compared to the other clusters (*p* < 0.001). NC6 (M = 30.77, SD = 6.37) also endorsed significantly more positive words than NC2 (M = 23.70, SD = 3.72), NC3 (M = 13.40, SD = 3.65), NC4 (M = 26.00, SD = 4.09), NC5 (M = 14.62, SD = 4.72, *p* < 0.01). Similarly, NC4 endorsed significantly more positive words than NC3, NC5, NC7 (M = 9.25, SD = 3.86, *p* < 0.001). Conversely, NC7 endorsed significantly fewer positive words than the other clusters (*p* < 0.001). NC3 also endorsed significantly fewer positive words than NC2, NC4, NC6 (*p* < 0.001). Similarly, NC5 also endorsed significantly fewer positive words than NC1, NC2, NC4 (*p* < 0.001). See Supplementary Appendix 4 for pairwise comparisons of endorsement rates across all samples.

### Cross-cluster difference In reaction time of endorsement of positive/ negative words

One-way between-subject ANOVA was also performed to examine the effect of clusters on reaction time in response to positive and negative words.

#### Clinical clusters

Clusters significantly predicted reaction time to positive words (*F* (4, 111) = 3.84, *p* = 0.006, *η*^2^ = 0.12; see Table 5) and also to negative words (*F* (4, 112) = 6.80, *p* < 0.001, *η*^2^ = 0.20; see Table 6) in the clinical sample. Post-hoc analysis using Tukey’s HSD revealed that individuals in C5 (M = 0.13, SD = 0.42) had a significantly slower reaction time to endorse negative words compared to individuals in C4 (M = −0.68, SD = 1.13, 95%CI [−1.47, −0.15], *p* = 0.008).

**Table 5:** Analysis of variance (ANOVA) results for the effect of clusters on reaction time to positive words.

Source	Sum of squares	*df*	Mean square	*F*	*η* ^2^	*p*
Clinical						
Cluster	10.40	4	2.600	3.84	0.12	0.006**
Residuals	75.07	111	0.68			
Non-clinical						
Cluster	1.87	4	0.47	2.83	0.10	0.03*
Residuals	16.97	103	0.16			
Combined						
Cluster	20.87	6	3.48	7.16	0.16	<0.001***
Residuals	107.38	221	0.49			

*p < 0.05, **p < 0.01, ***p < 0.001.

**Table 6:** Analysis of variance (ANOVA) results for the effect of clusters on reaction time to negative words.

Source	Sum of squares	*df*	Mean square	*F*	*η* ^2^	*p*
Clinical						
Cluster	7.87	4	1.97	6.80	0.20	<0.001***
Residuals	32.39	112	0.29			
Non-clinical						
Cluster	2.88	4	0.72	2.37	0.10	0.06
Residuals	32.56	107	0.30			
Combined						
Cluster	8.72	6	1.45	6.17	0.15	<0.001***
Residuals	51.35	218	0.24			

***p < 0.001.

Post-hoc tests also showed that individuals in C2 (M = −0.54, SD = 0.90) had a significantly faster reaction time to endorse positive words compared to those in C1 (M = 0.01, SD = 0.48, 95%CI [0.06, 1.03], *p* = 0.02). Similarly, individuals in C4 (M = 0.16, SD = 0.41) exhibited slower reaction time endorsing positive words when compared to C2 (95%CI [−1.06, −0.014], *p* < 0.001).

#### Non-clinical clusters

Clusters also significantly predicted reaction time to endorsing positive words in the non-clinical sample (*F* (4, 103) = 2.83, *p* = 0.03, *η*^2^ = 0.10; see Table 5). Post-hoc tests revealed that individuals in N4 (M = −0.09, SD = 0.39) had a slower reaction time endorsing positive words than individuals in N1 (M = −0.48, SD = 0.64).

#### Combined clusters

Furthermore, clusters also significantly predicted reaction time for endorsing both positive words (*F* (6, 221) = 7.16, *p* < 0.001, *η*^2^ = 0.16; see Table 5) and negative words (*F* (6, 218) = 6.17, *p* < 0.001, *η*^2^ = 0.15; see Table 6) in the combined sample. Post-hoc tests revealed that NC3 (M = 0.12, SD = 0.46) had a slower reaction time endorsing positive words compared to NC1 (M = −0.41, SD = 0.54, 95%CI [−0.54, 0.25], *p* = 0.003). Similarly, NC7 (M = 0.11, SD = 0.41) had a slower reaction time to positive words compared to NC1 (95%CI [−0.84, −0.19], *p* < 0.001); NC2 (M = −0.27, SD = 0.29, 95%CI [−0.74, −0.01], *p* = 0.04) but had a faster reaction time to positive words than NC6 (M = −0.51, SD = 0.74, 95%[−1.10,−0.14], *p* = 0.003) NC6 had a faster reaction time to positive words than NC3 (M = 0.12, SD = 0.46, 95%CI [0.08, 1.17], *p* = 0.01).

In addition, NC7 (M = −0.67, SD = 1.10) had a faster reaction time for endorsing negative words than NC1 (M = 0.17, SD = 0.68, 95%CI [0.39, 1.30], *p* < 0.001); NC2 (M = −0.08, SD = 0.28, 95%CI [0.07, 1.12], *p* = 0.02); NC4 (M = 0.08, SD = 0.28, 95%CI [0.33, 1.18], *p* < 0.001); NC5 (M = −0.13, SD = 0.55, 95%CI [0.10, 0.98], *p* = 0.005) and NC6 (M = 0.06, SD = 0.72, 95%CI [0.08, 1.38], *p* = 0.02). See Supplementary Appendix 5 for pairwise comparisons of reaction time bias across all samples.

### Cross-cluster difference in recall bias

One-way between-subject ANOVA was also performed to examine the effect of clusters on recall bias.

#### Clinical clusters

The results show that clusters significantly predicted negative recall bias in the clinical group (*F* (4, 114) = 3.55, *p* = 0.009, *η*^2^ = 0.11; see Table 7). Tukey’s HSD *post hoc* tests were performed to examine group differences. Notably, we applied a value of *p* threshold of 0.1 for interpretation, allowing us to emphasize practical significance. This choice was made to reduce the risk of Type I errors and align with the exploratory nature of this study. Using this threshold, individuals in C4 (M = 0.11, SD = 0.08) had a weaker recall bias for negative words as compared to C2 (M = 0.19, SD = 0.10, 95%CI [−0.001, 0.15], *p* = 0.06). Similarly, individuals in C5 (M = 0.10, SD = 0.12) also had a weaker recall bias for negative words as compared to C2 (95%CI [−0.001, 0.15], *p* = 0.06).

**Table 7:** Analysis of variance (ANOVA) results for the effect of clusters on recall bias in all samples.

Source	Sum of squares	*df*	Mean square	*F*	*η* ^2^	*p*
**Clinical**						
Negative recall bias						
Cluster	0.18	4	0.04	3.55	0.11	0.009*
Residuals	1.43	114	0.01			
**Combined**						
Positive recall bias						
Cluster	0.25	6	0.041	2.21	0.056	0.04
Residuals	4.21	227	0.019			
Negative recall bias minus positive recall bias						
Cluster	0.37	6	0.061	2.22	0.056	0.04
Residuals	6.19	225	0.027			

*p < 0.05.

#### Combined clusters

On the other hand, clusters significantly predicted positive recall bias (*F* (6, 227) = 2.21, *p* = 0.04, *η*^2^ = 0.06; see Table 7) and the difference between negative recall bias and positive recall bias (*F* (6, 225) = 2.22, *p* = 0.04, *η*^2^ = 0.06; see Table 7) between the combined clusters. While Tukey’s HSD did not reveal a significant difference between the combined clusters for positive recall bias, it did reveal a significant difference in terms of the difference between negative recall bias and positive recall bias. Specifically, NC1 (M = −0.10, SD = 0.17) exhibited a stronger memory bias towards positive words than negative words, as compared to NC7 who exhibited a stronger memory bias towards negative words (M = 0.007, SD = 0.15, 95%CI [−0.22, −0.001], *p* = 0.045). See Supplementary Appendix 6 for pairwise comparisons of recall bias across all samples.

**Table 8:** Analysis of variance (ANOVA) results for the effect of clusters on depressive scores (IDS-30) on all samples.

Source	Sum of squares	*df*	Mean square	*F*	*η* ^2^	*p*
Clinical						
Cluster	2770.69	4	692.67	3.94	0.12	0.005*
Residuals	20037.75	114	175.77			
Non-clinical						
Cluster	944.90	4	236.23	1.81	0.06	0.13
Residuals	14371.48	110	130.65			
Combined						
Cluster	17458.82	6	2909.80	17.87	0.32	<0.001**
Residuals	36967.67	227	162.85			

*p < 0.05, **p < 0.001.

### Cross-cluster difference in depressive symptoms

One-way between-subject ANOVA revealed that clusters significantly predicted depressive symptom severity in the clinical group (F (4, 114) = 3.94, *p* = 0.0054, *η*^2^ = 0.12; see Table 8). Post-hoc analysis using Tukey HSD revealed that among the clinical clusters, people in C5 (M = 20, SD = 9.40) had significantly less depressive symptoms compared to those in C1 (M = 37.4, SD = 10, 95%CI [−30.21, −4.61], *p* = 0.002) and C4 (M = 32.7, SD = 14.8, 95%CI [−23.26, −2.09], *p* = 0.01). None of the other clusters were significantly different from one another in depressive symptoms (*p* > 0.06).

One-way between-subject ANOVA revealed that clusters did not predict depressive symptoms in the non-clinical group (*F* (4, 110) = 1.81, *p* = 0.13, *η*^2^ = 0.06; see Table 8).

**Figure 4:**
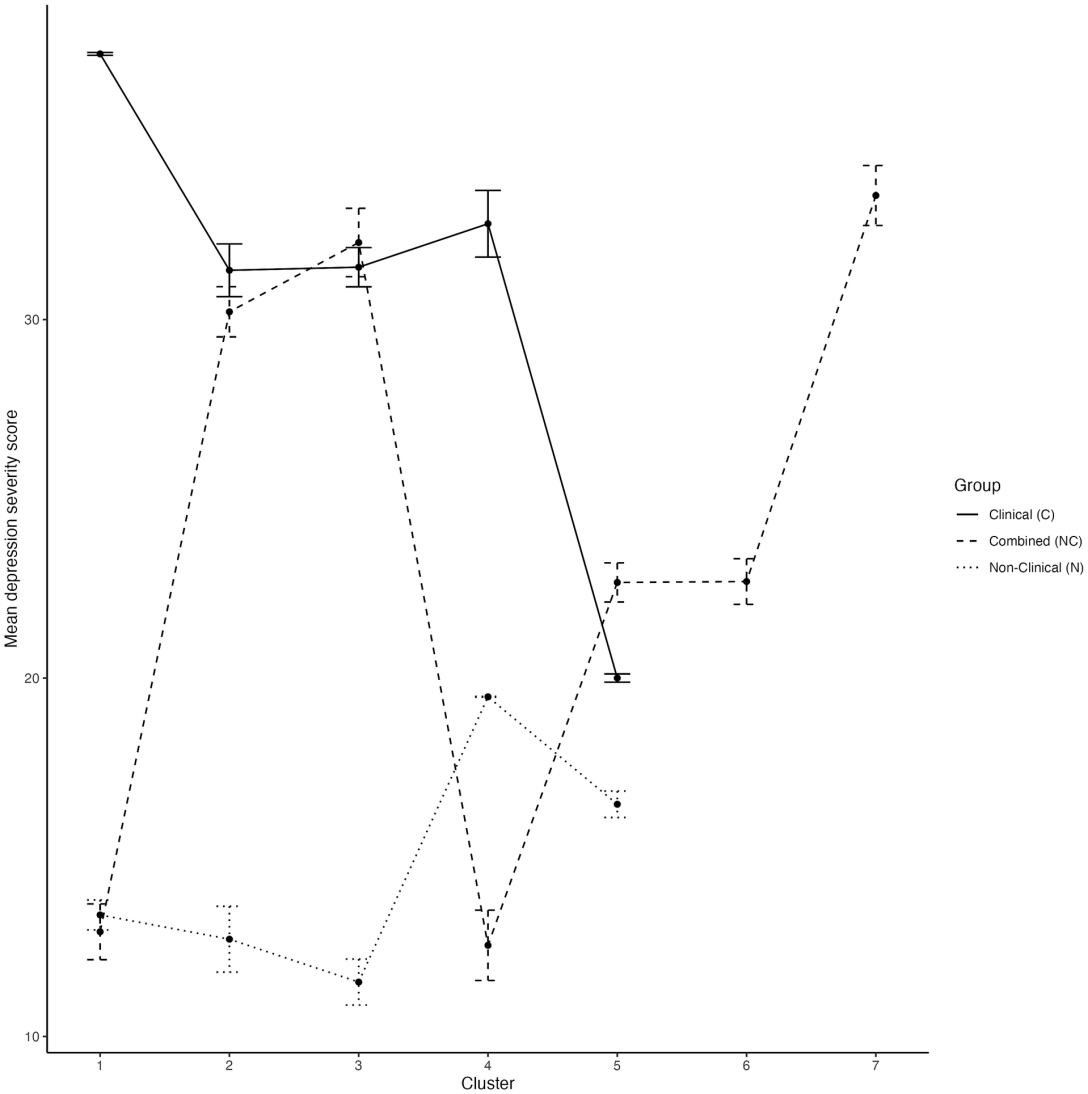
Depression symptom severity differences (IDS-30 scores) across all clusters. Error bars denote the standard error of the mean.

One-way between-subject ANOVA also revealed that clusters significantly predicted depressive symptom severity in the combined group (F (6, 227) = 17.87, *p* < 0.001, *η*^2^ = 0.32; see Table 8). For a visual representation of the differences in symptom severity in all samples, please refer to the line graph in Figure 4. Post-hoc tests showed that people in NC1 (M = 12.92, SD = 11.21) had significantly less depressive symptoms compared to people in NC2 (M = 30.22, SD = 16.17, 95%CI [−27.33, −7.27], *p* < 0.001), NC3 (M = 32.15, SD =12.35, 95%CI [−29.72, −8.74], *p* < 0.001) and NC7 (M = 33.46, SD =14.67, 95%CI [−28.64, −12.44], *p* < 0.001). Additionally, it was found that NC2 had significantly more depressive symptoms compared to NC4 (M = 12.54, SD =10.78, 95%CI [7.98, 27.37], *p* < 0.001), while NC3 also exhibited significantly more depressive symptoms compared to NC4 (M = 12.54, SD =10.78, 95%CI [9.44, 29.78], *p* < 0.001). Moreover, NC4 also had significantly less depressive symptoms in comparison to NC7 (M = 33.46, SD =14.67, 95%CI [−28.60, −13.23], *p* < 0.001). See Supplementary Appendix 7 for pairwise comparisons of depressive symptoms across all samples.

### Cross-cluster difference in depression subtypes among clinical clusters

Since RecDEQ data was available in 85 clinical participants, one-way ANOVAs were conducted to examine if the clustering solution distinguished between the dependency and self-critical subtypes of depression. Clinical clusters did not differ in dependency scores (*F* (4, 80) = 1.22, *p* = 0.31, *η*^2^ = 0.06) but they significantly differed in self-criticism scores (F (4, 80) = 3.10, *p* = 0.02, *η*^2^ = 0.13). Specifically, C1 (Neurotic) (M = 48.8, SD = 7.72, 95%CI [−19.74, −0.49], *p* = 0.035) and C4 (Self-critical) (M = 47.7, SD = 7.31, 95%CI [−17.04, −0.88], *p* = 0.022) were higher on self-criticism than C5 (Conscientious) (M = 38.7, SD = 12.4), corresponding to the difference in their depressive symptoms. Refer to Supplementary Figure S2 for graphical representation of dependency and self-criticism of the clinical clusters.

### Distribution of psychiatric diagnoses between clinical clusters

Among diagnostic categories, only Depressive Disorders showed significant differences in frequency between clusters (*χ*^2^(4) = 34.70, *p* < 0.001; see Table 9). C1 and C4 appeared to have higher proportions of individuals with depressive disorders, while C3 and C5 had lower proportions of individuals with depressive disorders.

**Table 9:** Diagnostic characteristics of participants in the five clinical clusters.

	Clinical sample (*N* = 119)				
	C1 (Anxious) (%) *N* = 17	C2 (Extraverted) (%) *N* = 24	C3 (Ambivalent) (%) *N* = 13	C4 (Self-critical) (%) *N* = 49	C5 (Non-neurotic and conscientious) (%) *N* = 16
Diagnoses					
**Descriptive statistics**					
Anxiety/anxiety-depression	4 (23.5%)	4 (16.7%)	2 (15.4%)	9 (18.4%)	4 (25%)
Depression/anxiety-depression	13 (76.5%)	13 (54.2%)	5 (38.5%)	33 (67.3%)	7 (43.8%)
Adjustment disorder	1 (5.9%)	6 (25%)	5 (38.5%)	6 (12.2%)	3 (18.8%)
Bipolar disorder	1 (5.9%)	1 (4.2%)	1 (7.7%)	3 (6.1%)	1 (6.3%)
Obsessive compulsive disorder (OCD, OCD with depression, OCD with Tourette’s, OCD with anxiety and depression)	1 (5.9%)	0 (0%)	1 (7.7%)	3 (6.1%)	1 (6.3%)
Additional comorbid psychiatric diagnoses (schizophrenia, cluster B traits, Eating disorder, obsessive-compulsive personality disorder, gambling, alcohol dependence, substance abuse, insomnia, attention deficit disorder with hyperactivity)	0 (0%)	2 (8.3%)	2 (15.4%)	3 (6.1%)	3 (18.8%)
**Inferential statistics multinomial test**	*χ*^2^(4)				
Anxiety/anxiety-depression	5.91				
Depression/anxiety-depression	34.70^***^				
Adjustment disorder	4.48				

Distribution of diagnoses amongst the clinical clusters. Multinomial test found that only Depressive Disorders significantly differed in frequency between the clusters. ***p < 0.001.

## Discussion

While grouping individuals by SRJs has been applied in the field of personality (Scully and Terry, 2011), it has not been applied clinically to depression despite evidence that SRJs have important prognostic value (Nejad et al., 2013; LeMoult et al., 2017) both from the perspectives of self-referential processing and psychodynamic constructs of depression. In applying clustering of SRJs across clinical and non-clinical populations, our findings may further our understanding of how these very different theoretical frameworks relate to one another.

Metrics such as optimum cluster number, criterion value, likelihood, DBI and CHI remained relatively stable when perturbed by varying the seed used to initialize clustering and this reflected our observations that cluster centers also remained relatively stable.

Traits from the FFM, psychodynamic constructs and anxiety appeared to inform the most endorsed words in each cluster. Within the clinical clustering solution, five-factor adjectives such as “neurotic” in C1 and N4, “extraverted” in C2 and “conscientious” in C5 appeared among the most endorsed words. The link between the FFM and depression has been well-explored, with common findings of high neuroticism, low conscientiousness and low extraversion being personality traits strongly associated with depression (Malouff and Thorsteinsson, 2005; Kotov et al., 2010; Grav et al., 2012).

“Self-critical” clusters C4 and NC7 can also be understood in terms of Blatt’s introjective subtype of depression, which emphasizes self-criticism and an internalized focus, that also corresponds to endorsement of words for neuroticism and negative self-referential processing. Meanwhile, the “Anxious to please” cluster, C3, could be understood in terms of Blatt’s anaclitic subtype, which emphasizes dependency and a strong desire for external validation in individuals experiencing depressive symptoms, as they endorsed words related to agreeableness and introversion(Blatt and Zuroff, 1992; Grav et al., 2012; Marfoli et al., 2021).

Among clusters that appeared to show consistency in the combined solution were unique endorsement patterns between clinical and nonclinical solutions, such as self-critical NC7/ C4 clusters and self-confident NC1/N1 clusters and common endorsement patterns, such as NC2 (Low Endorsement)/ C2 (Extraverted)/ C1(Neurotic)/ N4 (Neurotic). The unique patterns may relate to patterns of self-schema that may be either absent in or protective against depression or associated with subtypes of depression not present in the normal population. The common ones could reflect overlap in underlying self-schema across both clinical and non-clinical populations.

Overall, clusters endorsing more negative words also tended to endorse fewer positive words, showed more negative biases in reaction time and negative recall bias, reported more severe depressive symptoms and a higher frequency of depressive disorders and more self-criticism in the clinical population. Previous studies have found that depressive symptoms and depressive disorders are predicted by negative self-referential processing during similar tasks (Beevers et al., 2019).

C1 (Neurotic) and C4 (Self-critical) members endorsed more negative words and had more severe depressive symptoms than the other clusters and reported higher introjection/self-criticism scores than C5 (Conscientious). C4 (Self-critical) members also endorsed fewer positive words than C1 (Neurotic) and were slower when endorsing positive words compared to those in C2 (Extraverted). Taken together, the results show that the C4 (Self-critical) members displayed a heightened propensity for negative self-referential processing and a deficit in endorsing positive SRJs. These findings are consistent with network analysis research that depressed individuals with self-critical views tend to maintain highly interconnected negative self-perceptions while undervaluing their positive self-schemas (Collins et al., 2021). C5 (Conscientious) emerged as the least depressed clinical cluster and endorsed the least negative words. C5 endorsed fewer positive words, but demonstrated a weaker negative recall bias than C2 (Extraverted). This is in agreement with a previous finding that low extraversion and low conscientiousness predict the development of depressive symptoms (Hakulinen et al., 2015; Jourdy and Petot, 2017).

Although there were no differences in depression symptom severity between non-clinical clusters, N4 (Neurotic) and N5 (High endorsement) members endorsed more negative self-schema and N4 members had slower RTs in positive self-evaluations compared to their self-confident counterparts in N1. This aligns with other studies showing more severely depressed patients also exhibited slower RTs when endorsing positive words, distinguishing them from nondepressed individuals (Collins and Winer, 2023). Conversely, N1 (Self-confident) members endorsed more positive words compared to all other clusters, indicating that individuals with greater self-confidence tend to perceive themselves more positively. This positive self-perception contributes to their overall psychological well-being. Numerous studies have demonstrated that positive self-esteem acts as a protective buffer against negative influences (Mann et al., 2004).

In the combined sample, NC2 (Externalising), NC3 (Neurotic), and NC7 (Self-critical) clusters exhibited higher levels of depressive symptoms when compared to the NC4 (Secure) cluster. Additionally, both NC2 and NC7 had elevated depressive symptoms scores than NC1 (Self-confident). Interestingly, NC2 tended to endorse more positive words than NC3 and NC7. Prior research has found that more aggressive groups of children demonstrated similar levels of positive-self-perception and did not differ in the number of positive words they endorsed as compared to children in the control group (Burgess and Younger, 2006). Hence, this points at a unique cognitive pattern of the externalizing cluster where they endorsed more positive self-referential words, that is distinct from the neurotic and self-critical clusters.

NC3 (Neurotic) comprised C1 (Neurotic) and N4 (Neurotic) suggesting consistency as a construct across populations. Both NC3 (Neurotic) and NC7 (Self-critical) endorsed fewer positive words and responded more slowly when endorsing positive words compared to NC1, indicating difficulties in making positive SRJs, but NC7 additionally had a faster RT endorsing negative words than NC1, which suggests that self-critical individuals may have a heightened awareness of negative self-referential information and may readily endorse such negative self-attributes.

We have identified clusters on the basis of SRJs using words that are meaningful across theoretical frameworks from personality, psychodynamic concepts of relatedness and self-definition, and self-referential processing with key distinctions that may be useful for further study both in healthy populations and clinically. While positive and negative self-referential processing is typically highly correlated, identifying subgroups where they differ may be clinically meaningful by providing targets for interventions focused on positive psychology or anhedonia (Sandman and Craske, 2021). Further work could characterize further differences in clinical characteristics and interpersonal patterns.

We also considered various limitations of our approach. Firstly, the clinical and non-clinical participants were obtained from different datasets and not matched on demographic and psychiatric characteristics, resulting in heterogeneity between groups. Secondly, there is substantial heterogeneity in terms of clinical diagnosis within the clinical group. Further, the clustering analyses may be limited in their generalizability due to the relatively small sample sizes. Our ability to cluster using additional data such as recall bias or latency was limited by the sparse nature or small number of measures that could be derived from them, however it would be useful to develop ways to integrate such data into our clustering approach. It could also be useful to incorporate neuroimaging or EEG data in conjunction with behavioral data from SRET. While behavioral data can reveal overt manifestations of these conditions, it is often limited in its ability to uncover underlying neural mechanisms. The incorporation of neuroimaging data provides a means to directly visualize and measure brain activity and structure, helping us go beyond mere classification to uncover the neural signatures that differentiate healthy and affected individuals. Moreover, such approaches have been used in the classification of depression and other psychiatric conditions using deep learning methods such as Convolutional Neural Networks (CNNs), which have shown promise in classification of psychiatric conditions (Strambo et al., 2018; Ke et al., 2019, 2020, 2022, 2023).

The study was not designed to test the directionality of the relationship between SRP/self-concepts and depression. While negative self-schemas and SRP biases are posited to be stable individual characteristics that precede depression and can confer risk to depression (Beck, 1967; Derry and Kuiper, 1981), in reality, the relationship between them is more complex and bidirectional. For instance, Hayden et al. (2013) found that while negative and positive SRP prospectively predicted depressive symptoms in a community sample of children, depressive symptoms also prospectively predicted their negative SRP. As such, it is uncertain whether the current association found between self-concept-based clusters and depressive symptoms is due to such self-concepts contributing to the experience of depressive symptoms, or depressive moods leading to the development of certain self-concepts. It is also uncertain whether currently found self-endorsement clusters will remain stable after participants’ depressive symptoms subside, highlighting the importance of longitudinal studies to investigate the directionality of relationships and stability of self-concept subtypes linked to depression.

SRJ-based clustering represents a novel transdiagnostic framework for subgrouping patients with depressive and anxiety symptoms that may support the future translation of science of self-referential processing, personality and psychodynamic concepts of self-definition to clinical applications.

## Data Availability

The raw data supporting the conclusions of this article will be made available by the authors, without undue reservation.
