# Correction: Transdiagnostic clustering of self-schema from self-referential judgements identifies subtypes of healthy personality and depression

**DOI:** 10.3389/fninf.2025.1633196

**Published:** 2025-07-31

**Authors:** Geoffrey Chern-Yee Tan, Ziying Wang, Ethel Siew Ee Tan, Rachel Jing Min Ong, Pei En Ooi, Danan Lee, Nikita Rane, Sheryl Yu Xuan Tey, Si Ying Chua, Nicole Goh, Glynis Weibin Lam, Atlanta Chakraborty, Anthony Khye Loong Yew, Sin Kee Ong, Jin Lin Kee, Xin Ying Lim, Nawal Hashim, Sharon Huixian Lu, Michael Meany, Serenella Tolomeo, Christopher Lee Asplund, Hong Ming Tan, Jussi Keppo

**Affiliations:** ^1^Institute of Mental Health, Singapore, Singapore; ^2^Yale-NUS College, Singapore, Singapore; ^3^Faculty of Social Sciences, National University of Singapore, Singapore, Singapore; ^4^School of Biological Sciences, National Technological University, Singapore, Singapore; ^5^KK Women's and Children's Hospital, Singapore, Singapore; ^6^Institute of Operations Research and Analytics, National University of Singapore, Singapore, Singapore; ^7^NHG Polyclinics, Singapore, Singapore; ^8^Ministry of Education, Singapore, Singapore; ^9^Singapore Institute for Clinical Sciences, A^*^STAR, Singapore, Singapore; ^10^Institute of High Performance Computing (IHPC), Agency for Science, Technology and Research (A^*^STAR), Singapore, Singapore

**Keywords:** self-schema, self-concept, self-referential processing, personality, depression, depression subtypes, clustering

In the published article, there was an error in the author list, article citation and copyright section where the author name “Christopher Lee Asplund” was incorrectly listed as “Christopher Asplund Lee”. The correct version should have been “Christopher Lee Asplund”, “Asplund CL” and “Asplund” respectively in these sections.

In the published article, there was an error in the Funding statement. The Funding statement was erroneously presented as: “This research was supported by A^*^STAR Brain–Body Initiative (BBI) (#21718), IMH Research Seed Funding (642–2018), LKCMed-NUSMed-NHG Collaborative Mental Health Research Pilot Grant Call 2020 (MHRPG/2003) and FY21 PRENATAL/EARLY CHILDHOOD GRANT CALL (H22P0M0005).” The corrected Funding statement appears below.

