# Supplementary material for: Transdiagnostic clustering of self-schema from self-referential judgements identifies subtypes of healthy personality and depression
Source: Front Neuroinform. 2024 Jan 11;17:1244347. doi: 10.3389/fninf.2023.1244347 (PMC10808829; doi:10.3389/fninf.2023.1244347)
Supplement: Supplementary file 1 [file Table_1.DOCX]

***Supplementary Material***

## **2. Supplementary Tables**

## **TABLE A1 |** Demographic and Clinical Characteristics of Clinical and Non-clinical Groups

|  | Xchange  (N=85) | | Choldep patient  (N=34) | | Choldep control  (N=18) | | Undergraduate  (N=97) | |
| --- | --- | --- | --- | --- | --- | --- | --- | --- |
| Demographic Characteristics |  |  |  |  |  |  |  |  |
|  | Mean | SD | Mean | SD | Mean | SD | Mean | SD |
| Mean Age (SD) | 33.52 | 12.05 | 29.53 | 8.05 | 27.44 | 6.38 | 23.45 | 2.56 |
|  | n | % | n | % | n | % | n | % |
| Sex |  |  |  |  |  |  |  |  |
| Male | 41 | 48.2 | 19 | 55.9 | 9 | 50 | 30 | 30.9 |
| Female | 44 | 51.8 | 15 | 44.1 | 9 | 50 | 67 | 69.1 |
| Ethnicity |  |  |  |  |  |  |  |  |
| Chinese | 71 | 83.5 | 24 | 70.6 | 16 | 89 | 85 | 87.6 |
| Malay | 5 | 5.9 | 3 | 8.8 | 1 | 5.5 | 2 | 2.1 |
| Indian | 3 | 3.5 | 5 | 14.7 | 0 | 0 | 5 | 5.2 |
| Others | 6 | 7.1 | 2 | 5.9 | 1 | 5.5 | 5 | 5.2 |
| Clinical characteristics |  |  |  |  |  |  |  |  |
|  | Mean | SD | Mean | SD | Mean | SD | Mean | SD |
| Depressive symptoms |  |  |  |  |  |  |  |  |
| IDS-30-SR | 31.53 | 12.19 | 30.56 | 17.66 | 11.17 | 13.30 | 15.19 | 11.21 |
|  | n |  |  | % | n |  |  | % |
| Depressive category based on IDRS-30-SR cut-off score (Symptomatic $\geq$ 18) | | | | | | |  |  |
| Symptomatic | 71 | 83.5 | 24 | 70.6 | 3 | 16.7 | 37 | 38.1 |
| Non-symptomatic | 14 | 16.5 | 10 | 29.4 | 15 | 83.3 | 60 | 61.9 |
| Have had or currently have anxiety and/or depression symptoms? | | | | | | |  |  |
| Yes | 85 | 100 | 34 | 100 | 0 | 0 | 2 | 2.1 |
| No | - |  |  | - | 6 | 33.3 | 94 | 96.9 |
| Missing Data  Diagnoses in clinical sample | - |  |  | - | 12 | 66.7 | 1 | 1 |

## 
