# Supplementary material for: Transdiagnostic clustering of self-schema from self-referential judgements identifies subtypes of healthy personality and depression
Source: Front Neuroinform. 2024 Jan 11;17:1244347. doi: 10.3389/fninf.2023.1244347 (PMC10808829; doi:10.3389/fninf.2023.1244347)
Supplement: Supplementary file 2 [file Table_2.DOCX]

***Supplementary Material***

**TABLE A2 |** Correlations Between Age and SRET Variables with Depressive Symptoms in Clinical, Non-clinical, and Overall Samples

| Variable | Clinical | Non-clinical | Overall |
| --- | --- | --- | --- |
| Age | -0.03 | 0.01 | 0.23*** |
| Number of Negative words endorsed | 0.30*** | 0.24** | 0.48*** |
| Number of Positive words endorsed | -0.15 | -0.09 | -0.30*** |
| Negative RT Bias | -0.15 | -0.08 | -0.23*** |
| Positive RT Bias | 0.13 | 0.17 | 0.18** |
| Negative Recall Bias | 0.19 | 0.15 | 0.31*** |
| **p* $\leq$ .05. ***p* $\leq$ .01. ****p* $\leq$ .001. | | | |
| *Note.* R coefficient values are presented in the table | | | |
