# Supplementary material for: Transdiagnostic clustering of self-schema from self-referential judgements identifies subtypes of healthy personality and depression
Source: Front Neuroinform. 2024 Jan 11;17:1244347. doi: 10.3389/fninf.2023.1244347 (PMC10808829; doi:10.3389/fninf.2023.1244347)
Supplement: Supplementary file 3 [file Table_3.DOCX]

***Supplementary Material***

## **Supplementary Data**

## **Appendix A**

90 Common SRET Words for Clustering Across Three Samples

| Positive (count: 42) | Negative (count: 48) |
| --- | --- |
| at ease  attractive  brave  calm  cheerful  curious  efficient  enthusiastic  exciting  friendly  funny  happy  healthy  helpful  imaginative  inquisitive  interesting  leader  lively  loved  lucky  neighbourly  nice  orderly  organised  persistent  popular  proud  reflective  relaxed  reliable  secure  self-assured  self-confident  smart  stable  successful  systematic  unnervous  unworrying  well-off  winner | abandoned  accused  afraid  angry  anti-social  anxious  awful  bad  bashful  boring  burdened  chubby  coward  cruel  distant  empty  fretful  helpless  high-strung  hurt  hypersensitive  ill  impractical  inefficient  introverted  *lazy*  *lonely*  *loss*  *meek*  *neglected*  *nervous*  *quiet*  scared  shy  strange  stupid  tense  timid  tricky  ugly  uncharitable  uncheery  unkind  unsparkling  upset  useless  victim  worrying |
|  |  |
|  |  |
|  |  |
|  |  |
|  |  |
|  |  |
|  |  |
|  |  |
|  |  |
|  |  |
|  |  |
|  |  |
|  |  |
|  |  |
|  |  |
|  |  |
|  |  |
|  |  |
|  |  |
|  |  |
|  |  |
|  |  |
|  |  |
|  |  |
|  |  |
|  |  |
|  |  |
|  |  |
|  |  |
|  |  |
|  |  |
|  |  |
|  |  |
|  |  |
|  |  |
|  |  |
|  |  |
|  |  |
|  |  |
|  |  |
|  |  |
|  |  |
|  |  |
|  |  |
|  |  |
|  |  |
|  |  |
