# Supplementary material for: Transdiagnostic clustering of self-schema from self-referential judgements identifies subtypes of healthy personality and depression
Source: Front Neuroinform. 2024 Jan 11;17:1244347. doi: 10.3389/fninf.2023.1244347 (PMC10808829; doi:10.3389/fninf.2023.1244347)
Supplement: Supplementary file 5 [file Table_5.DOCX]

***Supplementary Material***

**TABLE A11 |** Clinical Clusters: Mapping of Top-Endorsed Words to Personality.

|  | C1  Anxious  (N=17) | C2  Extraverted  (N=24) | C3  Ambivalent  (N=13) | C4  Self-critical  (N=49) | C5  Non-neurotic and conscientious  (N=16) |
| --- | --- | --- | --- | --- | --- |
| Extraversion | Introverted  Lonely  Quiet | **Brave**  Lively  Cheerful  **Enthusiastic**  Exciting  Happy | Cheerful  Lively  Introverted  Shy  Lonely  Quiet  **Timid** | Introverted  Lonely  Quiet  Shy | Quiet |
| Agreeableness | Helpful | Helpful  Nice  Friendly | Helpful  Nice  Friendly | Helpful  Nice  **Bad** | Helpful  Nice  Friendly |
| Conscientiousness | **Systematic**  Lazy | Reliable |  | Lazy | **Efficient**  Reliable |
| Neuroticism | Afraid  Anxious  Angry  Scared  **Tense**  Upset  Nervous  Worrying  Hypersensitive | Afraid  Anxious  Scared  Upset  Nervous | Afraid  Anxious  Scared  Nervous  Worrying  Hypersensitive | Afraid  Anxious  Angry  Scared  Nervous  Upset | **Calm**  Anxious |
| Openness To Experience | Curious | Curious  **Reflective** | Curious | Curious | Curious  **Inquisitive** |
| Dependency | Lonely  Empty  Hurt | Loved  Hurt | Loved  Lonely  Empty  Hurt | Hurt  **Abandoned** | Loved |
| Self-Criticism | Useless  Lazy  Boring  Strange  Helpless | Smart  **Interesting**  Funny  Strange | Funny  **Healthy**  Useless  Boring  Ugly  **Chubby** | Useless  Lazy  Boring  Helpless  Strange  Ugly  **Stupid**  **Coward**  **Bad** | Smart  Funny |
| Others | **Burdened** |  |  |  |  |

*Five clinical clusters were generated from clustering analysis. Clusters are labeled according to the traits they more often endorse. Top-endorsed words have a mean endorsement equal to or above the upper quartile of mean endorsements of words within a cluster. Words that are uniquely endorsed by one cluster but not the other clusters are bolded. When both negative-valence and positive-valence words are endorsed within any dimension, positive words are listed first, followed by negative words.*

**TABLE A12 |** Non-clinical Clusters: Mapping of Top-Endorsed Words to Personality.

|  | N1  (Self-confident)  (N=23) | N2  (Ambivalent)  (N=21) | N3  (Non-neurotic)  (N=27) | N4  (Introverted)  (N=21) | N5  (Conscientious)  (N=23) |
| --- | --- | --- | --- | --- | --- |
| Extraversion | Brave  Cheerful  Enthusiastic  Exciting  Happy  Lively  Self-Assured  **Self-Confident** | Cheerful  Happy  Persistent    Introverted  Quiet | Cheerful  Enthusiastic  Happy  Lively  Self-Assured | **Bashful**  **Distant**  Introverted  Quiet  Shy  **Timid** | Brave  Cheerful  Enthusiastic  Exciting  Happy  Lively  Persistent  **Proud**  Self-Assured  Introverted  Shy |
| Agreeableness | Helpful  Nice  Friendly | Helpful  Nice  Neighbourly  Friendly | Helpful  Nice  Neighbourly  Friendly | Helpful  Nice  Friendly | Helpful  Nice  Friendly |
| Conscientiousness | Reliable  Efficient  Systematic | Reliable  Efficient | Reliable  Efficient  Lazy | Reliable  Lazy | Efficient  Reliable  **Organized**  Systematic  **Orderly** |
| Neuroticism | Stable  Relaxed  At Ease  Calm | Calm  Stable  Anxious  Nervous  Worrying | Stable  Relaxed  At Ease  Calm | Anxious  Nervous  Scared  **Tense**  Worrying  **Fretful**  Afraid  Hypersensitive | Calm  Anxious  Nervous  Scared  Worrying  Afraid  Hypersensitive |
| Openness To Experience | Curious  Reflective  Inquisitive | Curious  Reflective  Inquisitive | Curious  Reflective | Curious  Reflective | Reflective  **Imaginative**  Curious |
| Dependency | Loved  Secure | Loved | Loved  Secure | Loved | Loved  Secure  **Hurt** |
| Self-Criticism | Smart  Interesting  Funny  Healthy  **Self-Confident** | Smart  Funny  Healthy  Strange  Boring | Interesting  Healthy | Boring  Strange | Funny  Strange |
| Others | Lucky |  | Lucky | **Burdened** |  |

*Five non-clinical clusters were generated from clustering analysis. Clusters are labeled according to the traits they more often endorse. Top-endorsed words have a mean endorsement equal to or above the upper quartile of mean endorsements of words within a cluster. Words that are uniquely endorsed by one cluster but not the other clusters are bolded.*

**TABLE A13 |** Combined Clinical and Non-clinical: Mapping of Top-Endorsed Words to Personality.

|  | NC1  (Self-confident)  (N=38) | NC2  (Externalising)  (N=23) | NC3  (Anaclitic)  (N=20) | NC4  (Conscientious)  (N=46) | NC5  (Quiet)  (N=42) | NC6  (Anxious)  (N=13) | NC7  (Self-critical)  (N=52) |
| --- | --- | --- | --- | --- | --- | --- | --- |
| Extraversion | Cheerful  **Enthusiastic**  Exciting  Happy  Lively  **Self-assured** | Brave  Cheerful  Exciting  Lively  Persistent  **Distant**  Introverted | Introverted  Quiet  Shy  **Timid** | Cheerful  Happy  Introverted  Quiet | Quiet | Brave  Exciting  Happy  Persistent  Introverted  Quiet  Shy | Introverted  Quiet  Shy |
| Agreeableness | Friendly  Helpful  Nice | Friendly  Helpful  Nice | Helpful  Nice | Friendly  Helpful  **Neighbourly**  Nice | Friendly  Helpful  Nice | Friendly  Helpful  Nice | Helpful  Nice  **Bad** |
| Conscientiousness | Reliable | Efficient |  | Efficient  **Organized**  Reliable  **Systematic** | Reliable | Efficient  Reliable |  |
| Neuroticism | **At ease**  Calm  Stable  Relaxed | Afraid  **Angry**  Anxious  Hypersensitive  Nervous  Scared  Tense  Worrying | Afraid  Anxious  **Fretful**  Hypersensitive  Nervous  Scared  Tense  Upset  Worrying | Calm  Relaxed  Stable    Anxious  Nervous | Afraid  Anxious  Nervous  Scared  Upset | Afraid  Anxious  Hypersensitive  Nervous  Tense  Upset  Worrying  Calm | Afraid  Anxious  Nervous  Scared  Upset |
| Openness To Experience | Curious  Inquisitive  Reflective | Curious  Imaginative  Inquisitive  Reflective | Curious  Reflective | Curious  Imaginative  Reflective | Curious  Inquisitive | Curious  Imaginative  Reflective | Curious |
| Dependency | Loved  Secure | Loved  Hurt | Loved  Empty  Hurt  Lonely  **Loss** | Loved  Secure | Loved  Lonely | Hurt  Lonely | **Abandoned**  Empty  Hurt  Lonely |
| Self-Criticism | Funny  Healthy  Interesting | Funny  Interesting  **Leader**  Smart  Strange | Boring  Helpless  Lazy  Strange | Healthy  Lazy | Smart  Boring  Strange | Funny  Interesting  Smart  **Chubby** | **Bad**  Boring  **Coward**  Helpless  Lazy  Strange  **Stupid**  **Ugly**  **Useless** |
| Others | Lucky | Lucky | Burdened |  |  | Burdened |  |

*Seven combined clusters were generated from clustering analysis. Clusters are labeled according to the traits they more often endorse. Top-endorsed words have a mean endorsement equal to or above the upper quartile of mean endorsements of words within a cluster. Words that are uniquely endorsed by one cluster but not the other clusters are bolded.*
