# Supplementary material for: Transdiagnostic clustering of self-schema from self-referential judgements identifies subtypes of healthy personality and depression
Source: Front Neuroinform. 2024 Jan 11;17:1244347. doi: 10.3389/fninf.2023.1244347 (PMC10808829; doi:10.3389/fninf.2023.1244347)
Supplement: Supplementary file 6 [file Table_6.DOCX]

***Supplementary Material***

**TABLE A14 |** Pairwise Comparisons of Endorsement Rate Across Clinical Clusters

| Comparison | Mean difference | SE | 95% CI | t | p |
| --- | --- | --- | --- | --- | --- |
| Negative Endorsement |  |  |  |  |  |
| cluster 2 - cluster 1 | -15.30 | 1.70 | -20.02 - -10.58 | -8.98 | < 0.001*** |
| cluster 3 - cluster 1 | -12.65 | 1.98 | -18.13 - -7.16 | -6.39 | < 0.001*** |
| cluster 4 - cluster 1 | -6.17 | 1.51 | -10.36 - -1.96 | -4.08 | < 0.001*** |
| cluster 5 - cluster 1 | -23.82 | 1.87 | -29.01 - -18.63 | -12.73 | < 0.001*** |
| cluster 3 - cluster 2 | 2.65 | 1.85 | -2.48 - -7.78 | 1.43 | 0.60 |
| cluster 4 - cluster 2 | 9.13 | 1.34 | 5.42 - 12.84 | 6.82 | < 0.001*** |
| cluster 5 - cluster 2 | -8.52 | 1.73 | -13.33 - 3.71 | -4.91 | < 0.001*** |
| cluster 4 - cluster 3 | 6.48 | 1.68 | 1.84 - 11.13 | 3.87 | 0.0017 ** |
| cluster 5 - cluster 3 | -11.17 | 2.01 | -16.73 - -5.61 | -5.57 | < 0.001*** |
| cluster 5 - cluster 4 | -17.65 | 1.55 | -21.94 - -13.36 | -11.41 | < 0.001*** |
| Positive Endorsement |  |  |  |  |  |
| cluster 2 - cluster 1 | 5.04 | 1.88 | -0.16 - 10.25 | 2.68 | 0.060 |
| cluster 3 - cluster 1 | -4.32 | 2.18 | -10.38 - 1.73 | -1.98 | 0.28 |
| cluster 4 - cluster 1 | -8.52 | 1.67 | -13.15 - -3.90 | -5.11 | < 0.001*** |
| cluster 5 - cluster 1 | -0.83 | 2.06 | -6.55 - -4.89 | -0.40 | 0.99 |
| cluster 3 - cluster 2 | -9.37 | 2.04 | -15.02 - -3.71 | -4.59 | < 0.001*** |
| cluster 4 - cluster 2 | -13.57 | 1.48 | -17.66 - -9.47 | -9.19 | < 0.001*** |
| cluster 5 - cluster 2 | -5.88 | 1.91 | -11.18 - -0.57 | -3.07 | 0.021 * |
| cluster 4 - cluster 3 | -4.20 | 1.85 | -9.33 - 0.93 | -2.27 | 0.16 |
| cluster 5 - cluster 3 | 2.49 | 2.21 | -2.65 - 9.63 | 1.58 | 0.51 |
| cluster 5 - cluster 4 | 7.69 | 1.71 | 2.96 - 12.42 | 4.51 | < 0.001*** |
| **p* $\leq$ *.05. **p* $\leq$ *.01. ***p* $\leq$ *.001.* | | | | | |

#

**TABLE A15 |** Pairwise Comparisons of Endorsement Rate Across Non-Clinical Clusters

| Comparison | Mean difference | SE | 95% CI | t | p |
| --- | --- | --- | --- | --- | --- |
| Negative Endorsement |  |  |  |  |  |
| cluster 2 - cluster 1 | 6.20 | 1.59 | 1.79 - 10.61 | 3.90 | 0.0015 ** |
| cluster 3 - cluster 1 | 2.47 | 1.49 | -1.68 - 6.61 | 1.65 | 0.47 |
| cluster 4 - cluster 1 | 21.49 | 1.59 | 17.08 - 25.89 | 13.53 | < 0.001*** |
| cluster 5 - cluster 1 | 16.74 | 1.55 | 12.43 - 21.04 | 10.78 | < 0.001*** |
| cluster 3 - cluster 2 | -3.74 | 1.53 | -7.98 - 0.51 | -2.44 | 0.11 |
| cluster 4 - cluster 2 | 15.29 | 1.62 | 10.78 - 19.79 | 9.41 | < 0.001*** |
| cluster 5 - cluster 2 | 10.54 | 1.59 | 6.13 - 14.94 | 6.63 | < 0.001*** |
| cluster 4 - cluster 3 | 19.02 | 1.53 | 14.77 - 23.27 | 12.42 | < 0.001*** |
| cluster 5 - cluster 3 | 14.27 | 1.49 | 10.13 - 18.42 | 9.56 | < 0.001*** |
| cluster 5 - cluster 4 | -4.75 | 1.59 | -9.15 - -0.34 | -2.99 | 0.28 * |
| Positive Endorsement |  |  |  |  |  |
| cluster 2 - cluster 1 | -15.55 | 1.46 | -19.59 - -11.52 | -10.69 | <0.001*** |
| cluster 3 - cluster 1 | -7.03 | 1.37 | -10.82 - -3.23 | -5.14 | <0.001*** |
| cluster 4 - cluster 1 | -18.41 | 1.46 | -22.45 - -14.37 | -12.65 | <0.001*** |
| cluster 5 - cluster 1 | -6.78 | 1.42 | -10.73 - -2.84 | -4.77 | <0.001*** |
| cluster 3 - cluster 2 | 8.52 | 1.40 | 4.63 - 12.41 | 6.08 | <0.001*** |
| cluster 4 - cluster 2 | -2.86 | 1.49 | -6.98 - 1.27 | -1.92 | 0.31 |
| cluster 5 - cluster 2 | 8.77 | 1.46 | 4.73 - 12.81 | 6.03 | <0.001*** |
| cluster 4 - cluster 3 | -11.38 | 1.40 | -15.27 - -7.49 | -8.11 | <0.001*** |
| cluster 5 - cluster 3 | 0.25 | 1.37 | -3.55 - 4.04 | 0.18 | 1.00 |
| cluster 5 - cluster 4 | 11.63 | 1.46 | 7.59 - 15.66 | 7.99 | <0.001*** |
| **p* $\leq$ *.05. **p* $\leq$ *.01. ***p* $\leq$ *.001.* | | | | | |

# **TABLE A16 |** Pairwise Comparisons of Endorsement Rates Across Combined Clusters

| Comparison | Mean difference | SE | 95% CI | t | p |
| --- | --- | --- | --- | --- | --- |
| Negative Endorsement |  |  |  |  |  |
| cluster 2 - cluster 1 | 13.16 | 1.41 | -17.36 -8.95 | -9.30 | <.001*** |
| cluster 3 - cluster 1 | 26.89 | 1.48 | -31.29 -22.5 | -18.19 | <.001*** |
| cluster 4 - cluster 1 | 7.50 | 1.17 | -10.99 -4.01 | -6.39 | <.001*** |
| cluster 5 - cluster 1 | 7.13 | 1.20 | -10.7 -3.57 | -5.95 | <.001*** |
| cluster 6 - cluster 1 | 23.05 | 1.72 | -28.17 -17.93 | -13.40 | <.001*** |
| cluster 7 - cluster 1 | 20.07 | 1.14 | -23.47 -16.67 | -17.57 | <.001*** |
| cluster 3 - cluster 2 | 13.74 | 1.64 | -18.61 -8.87 | -8.40 | <.001*** |
| cluster 4 - cluster 2 | -5.65 | 1.37 | 1.59 9.72 | 4.13 | <.001*** |
| cluster 5 - cluster 2 | -6.02 | 1.39 | 1.89 10.15 | 4.34 | <.001*** |
| cluster 6 - cluster 2 | 9.89 | 1.86 | -15.42 -4.37 | -5.33 | <.001*** |
| cluster 7 - cluster 2 | 6.91 | 1.34 | -10.9 -2.92 | -5.16 | <.001*** |
| cluster 4 - cluster 3 | -19.39 | 1.43 | 15.13 23.66 | 13.53 | <.001*** |
| cluster 5 - cluster 3 | -19.76 | 1.45 | 15.44 24.09 | 13.59 | <.001*** |
| cluster 6 - cluster 3 | -3.85 | 1.91 | -1.83 9.52 | 2.02 | 0.41 |
| cluster 7 - cluster 3 | -6.83 | 1.41 | 2.64 11.02 | 4.85 | <.001*** |
| cluster 5 - cluster 4 | -0.37 | 1.14 | -3.03 3.77 | 0.32 | 1.00 |
| cluster 6 - cluster 4 | 15.55 | 1.68 | -20.55 -10.54 | -9.25 | <.001*** |
| cluster 7 - cluster 4 | 12.56 | 1.08 | -15.79 -9.34 | -11.60 | <.001*** |
| cluster 6 - cluster 5 | 15.92 | 1.70 | -20.97 -10.86 | -9.37 | <.001*** |
| cluster 7 - cluster 5 | 12.93 | 1.11 | -16.24 -9.63 | -11.65 | <.001*** |
| cluster 7 - cluster 6 | 2.98 | 1.66 | -1.96 7.92 | 1.80 | 0.55 |
| Positive endorsement |  |  |  |  |  |
| cluster 2 - cluster 1 | -10.07 | 1.14 | 6.69 13.45 | 8.86 | < .001*** |
| cluster 3 - cluster 1 | -20.36 | 1.19 | 16.83 23.9 | 17.14 | < .001*** |
| cluster 4 - cluster 1 | -7.76 | 0.94 | 4.96 10.57 | 8.24 | < .001*** |
| cluster 5 - cluster 1 | -19.14 | 0.96 | 16.28 22.01 | 19.89 | < .001*** |
| cluster 6 - cluster 1 | -2.99 | 1.38 | -1.12 7.1 | 2.17 | 0.32 |
| cluster 7 - cluster 1 | -24.51 | 0.92 | 21.78 27.24 | 26.71 | < .001*** |
| cluster 3 - cluster 2 | -10.30 | 1.31 | 6.38 14.21 | 7.83 | < .001*** |
| cluster 4 - cluster 2 | 2.30 | 1.10 | -5.57 0.96 | -2.10 | 0.36 |
| cluster 5 - cluster 2 | -9.08 | 1.12 | 5.76 12.39 | 8.14 | < .001*** |
| cluster 6 - cluster 2 | 7.07 | 1.49 | -11.51 -2.64 | -4.74 | < .001*** |
| cluster 7 - cluster 2 | -14.45 | 1.08 | 11.24 17.65 | 13.42 | < .001*** |
| cluster 4 - cluster 3 | 12.60 | 1.15 | -16.03 -9.17 | -10.94 | < .001*** |
| cluster 5 - cluster 3 | 1.22 | 1.17 | -4.69 2.26 | -1.04 | 0.94 |
| cluster 6 - cluster 3 | 17.37 | 1.53 | -21.93 -12.81 | -11.34 | < .001*** |
| cluster 7 - cluster 3 | -4.15 | 1.13 | 0.78 7.52 | 3.67 | 5.57×10^-3^** |
| cluster 5 - cluster 4 | -11.38 | 0.92 | 8.65 14.11 | 12.40 | < .001*** |
| cluster 6 - cluster 4 | 4.77 | 1.35 | -8.79 -0.75 | -3.53 | 8.93×10^-3^** |
| cluster 7 - cluster 4 | -16.75 | 0.87 | 14.16 19.34 | 19.25 | < .001*** |
| cluster 6 - cluster 5 | 16.15 | 1.36 | -20.21 -12.09 | -11.83 | < .001*** |
| cluster 7 - cluster 5 | -5.37 | 0.89 | 2.72 8.02 | 6.02 | < .001*** |
| cluster 7 - cluster 6 | -21.52 | 1.33 | 17.55 25.49 | 16.14 | < .001*** |

****p* $\leq$ *.001.*
