# Supplementary material for: Transdiagnostic clustering of self-schema from self-referential judgements identifies subtypes of healthy personality and depression
Source: Front Neuroinform. 2024 Jan 11;17:1244347. doi: 10.3389/fninf.2023.1244347 (PMC10808829; doi:10.3389/fninf.2023.1244347)
Supplement: Supplementary file 7 [file Table_7.DOCX]

***Supplementary Material***

# **Table A17 |** Pairwise Comparisons of Reaction Time Bias to Endorsing Words Across Clinical Clusters

| Comparison | Mean difference | SE | 95% CI | t | p |
| --- | --- | --- | --- | --- | --- |
| Negative Words |  |  |  |  |  |
| cluster 2 - cluster 1 | -0.054 | 0.261 | -0.669 0.777 | 0.206 | 1.000 |
| cluster 3 - cluster 1 | -0.103 | 0.303 | -0.737 0.943 | 0.339 | 0.997 |
| cluster 4 - cluster 1 | -0.554 | 0.233 | -0.094 1.201 | 2.372 | 0.131 |
| cluster 5 - cluster 1 | 0.256 | 0.286 | -1.051 0.538 | -0.895 | 0.898 |
| cluster 3 - cluster 2 | -0.049 | 0.283 | -0.736 0.834 | 0.173 | 1.000 |
| cluster 4 - cluster 2 | -0.500 | 0.207 | -0.074 1.074 | 2.414 | 0.119 |
| cluster 5 - cluster 2 | 0.310 | 0.265 | -1.046 0.426 | -1.169 | 0.769 |
| cluster 4 - cluster 3 | -0.451 | 0.258 | -0.265 1.167 | 1.745 | 0.411 |
| cluster 5 - cluster 3 | 0.359 | 0.307 | -1.211 0.492 | -1.170 | 0.768 |
| cluster 5 - cluster 4 | 0.810 | 0.239 | -1.472 -0.148 | -3.394 | 0.008** |
| Positive Words |  |  |  |  |  |
| cluster 2 - cluster 1 | -0.544 | 0.174 | 0.062 1.025 | 3.133 | 0.018* |
| cluster 3 - cluster 1 | -0.177 | 0.201 | -0.379 0.734 | 0.883 | 0.903 |
| cluster 4 - cluster 1 | 0.147 | 0.155 | -0.578 0.283 | -0.948 | 0.878 |
| cluster 5 - cluster 1 | -0.108 | 0.190 | -0.419 0.635 | 0.567 | 0.980 |
| cluster 3 - cluster 2 | 0.366 | 0.185 | -0.880 0.147 | -1.978 | 0.283 |
| cluster 4 - cluster 2 | 0.691 | 0.134 | -1.064 -0.138 | -5.138 | < .001*** |
| cluster 5 - cluster 2 | 0.436 | 0.174 | -0.917 0.045 | -2.512 | 0.095 |
| cluster 4 - cluster 3 | 0.324 | 0.168 | -0.791 0.142 | -1.930 | 0.308 |
| cluster 5 - cluster 3 | 0.070 | 0.201 | -0.626 0.487 | -0.347 | 0.997 |
| cluster 5 - cluster 4 | -0.255 | 0.155 | -0.176 0.685 | 1.642 | 0.474 |

**p* $\leq$ .05. ***p* $\leq$ *.01.* ****p* $\leq$ .001.

# **Table A18 |** Pairwise Comparisons of Reaction Time Bias to Endorsing Positive Words Across Non-Clinical Clusters

| Comparison | Mean difference | SE | 95% CI | t | p |
| --- | --- | --- | --- | --- | --- |
| Positive Words |  |  |  |  |  |
| cluster 2 - cluster 1 | 0.37 | 0.13 | -0.73 1.91×10^-3^ | -2.76 | 0.05 |
| cluster 3 - cluster 1 | 0.24 | 0.13 | -0.59 0.11 | -1.90 | 0.32 |
| cluster 4 - cluster 1 | 0.40 | 0.13 | -0.76 -0.03 | -3.00 | 0.03* |
| cluster 5 - cluster 1 | 0.33 | 0.13 | -0.69 0.04 | -2.51 | 0.10 |
| cluster 3 - cluster 2 | -0.13 | 0.12 | -0.20 0.45 | 1.07 | 0.82 |
| cluster 4 - cluster 2 | 0.03 | 0.13 | -0.38 0.32 | -0.25 | 1.00 |
| cluster 5 - cluster 2 | -0.04 | 0.12 | -0.31 0.38 | 0.30 | 1.00 |
| cluster 4 - cluster 3 | 0.16 | 0.12 | -0.49 0.17 | -1.34 | 0.67 |
| cluster 5 - cluster 3 | 0.09 | 0.12 | -0.41 0.23 | -0.77 | 0.94 |
| cluster 5 - cluster 4 | -0.07 | 0.12 | -0.28 0.41 | 0.55 | 0.98 |

**p* $\leq$ .05.

# **Table A19 |** Pairwise Comparisons of Reaction Time Bias to Positive Words Across Combined Clusters

| Comparison | Mean difference | SE | 95% CI | t | p |
| --- | --- | --- | --- | --- | --- |
| cluster 2 - cluster 1 | 0.14 | 0.13 | -0.54 0.25 | -1.07 | 0.94 |
| cluster 3 - cluster 1 | 0.53 | 0.14 | -0.94 -0.11 | -3.81 | 3.44×10^-3^ ** |
| cluster 4 - cluster 1 | 0.25 | 0.11 | -0.58 0.08 | -2.22 | 0.29 |
| cluster 5 - cluster 1 | 0.25 | 0.11 | -0.59 0.09 | -2.17 | 0.32 |
| cluster 6 - cluster 1 | -0.10 | 0.17 | -0.41 0.60 | 0.58 | 1.00 |
| cluster 7 - cluster 1 | 0.52 | 0.11 | -0.84 -0.19 | -4.74 | < .001 *** |
| cluster 3 - cluster 2 | 0.38 | 0.15 | -0.83 0.06 | -2.59 | 0.13 |
| cluster 4 - cluster 2 | 0.11 | 0.12 | -0.47 0.26 | -0.85 | 0.98 |
| cluster 5 - cluster 2 | 0.10 | 0.13 | -0.48 0.27 | -0.83 | 0.98 |
| cluster 6 - cluster 2 | -0.24 | 0.18 | -0.29 0.77 | 1.35 | 0.83 |
| cluster 7 - cluster 2 | 0.38 | 0.12 | -0.74 -0.01 | -3.09 | 0.04 * |
| cluster 4 - cluster 3 | -0.28 | 0.13 | -0.11 0.67 | 2.14 | 0.33 |
| cluster 5 - cluster 3 | -0.28 | 0.13 | -0.11 0.67 | 2.12 | 0.35 |
| cluster 6 - cluster 3 | -0.62 | 0.18 | 0.08 1.17 | 3.43 | 0.01 * |
| cluster 7 - cluster 3 | -7.47×10^-3^ | 0.13 | -0.37 0.39 | 0.06 | 1.00 |
| cluster 5 - cluster 4 | -6.51×10^-4^ | 0.10 | -0.31 0.31 | 6.29×10^-3^ | 1.00 |
| cluster 6 - cluster 4 | 0.35 | 0.16 | -0.14 0.83 | 2.12 | 0.34 |
| cluster 7 - cluster 4 | -0.27 | 0.10 | -0.56 0.02 | -2.75 | 0.09 |
| cluster 6 - cluster 5 | 0.35 | 0.16 | -0.14 0.83 | 2.10 | 0.36 |
| cluster 7 - cluster 5 | -0.27 | 0.10 | -0.57 0.03 | -2.69 | 0.11 |
| cluster 7 - cluster 6 | -0.62 | 0.16 | -1.10 -0.14 | -3.82 | 3.22×10^-3^ ** |

**p* $\leq$ .05. ***p* $\leq$ .01. ****p* $\leq$ .001.

# **Table A20 |** Pairwise Comparisons of Reaction Time Bias to Endorsing Negative Words Across Combined Clusters

| Comparison | Mean difference | SE | 95% CI | t | p |
| --- | --- | --- | --- | --- | --- |
| cluster 2 - cluster 1 | -0.25 | 0.19 | -0.30 0.80 | 1.35 | 0.83 |
| cluster 3 - cluster 1 | -0.50 | 0.19 | -0.08 1.08 | 2.58 | 0.14 |
| cluster 4 - cluster 1 | -0.09 | 0.16 | -0.38 0.55 | 0.55 | 1.00 |
| cluster 5 - cluster 1 | -0.30 | 0.16 | -0.17 0.77 | 1.91 | 0.48 |
| cluster 6 - cluster 1 | -0.11 | 0.23 | -0.56 0.78 | 0.49 | 1.00 |
| cluster 7 - cluster 1 | -0.84 | 0.15 | 0.39 1.30 | 5.51 | < .001 *** |
| cluster 3 - cluster 2 | -0.25 | 0.21 | -0.38 0.88 | 1.18 | 0.90 |
| cluster 4 - cluster 2 | 0.16 | 0.18 | -0.70 0.37 | -0.92 | 0.97 |
| cluster 5 - cluster 2 | -0.05 | 0.18 | -0.49 0.59 | 0.28 | 1.00 |
| cluster 6 - cluster 2 | 0.14 | 0.24 | -0.86 0.58 | -0.58 | 1.00 |
| cluster 7 - cluster 2 | -0.59 | 0.18 | 0.07 1.12 | 3.36 | 0.02 * |
| cluster 4 - cluster 3 | 0.41 | 0.19 | -0.97 0.14 | -2.21 | 0.29 |
| cluster 5 - cluster 3 | 0.20 | 0.19 | -0.76 0.36 | -1.05 | 0.94 |
| cluster 6 - cluster 3 | 0.39 | 0.25 | -1.13 0.35 | -1.57 | 0.70 |
| cluster 7 - cluster 3 | -0.34 | 0.18 | -0.21 0.89 | 1.85 | 0.52 |
| cluster 5 - cluster 4 | -0.22 | 0.15 | -0.23 0.66 | 1.44 | 0.78 |
| cluster 6 - cluster 4 | -0.02 | 0.22 | -0.63 0.68 | 0.11 | 1.00 |
| cluster 7 - cluster 4 | -0.76 | 0.14 | 0.33 1.18 | 5.26 | < .001 *** |
| cluster 6 - cluster 5 | 0.19 | 0.22 | -0.85 0.47 | -0.86 | 0.98 |
| cluster 7 - cluster 5 | -0.54 | 0.15 | 0.10 0.98 | 3.69 | 5.18×10^-3^ ** |
| cluster 7 - cluster 6 | -0.73 | 0.22 | 0.08 1.38 | 3.36 | 0.02 * |

***p* $\leq$ .01. ****p* $\leq$ .001.
