# Supplementary material for: Transdiagnostic clustering of self-schema from self-referential judgements identifies subtypes of healthy personality and depression
Source: Front Neuroinform. 2024 Jan 11;17:1244347. doi: 10.3389/fninf.2023.1244347 (PMC10808829; doi:10.3389/fninf.2023.1244347)
Supplement: Supplementary file 8 [file Table_8.DOCX]

***Supplementary Material***

# **Table A21 |** Pairwise Comparisons of Negative Recall Bias Across Clinical Clusters

| Comparison | Mean difference | SE | 95% CI | t | p |
| --- | --- | --- | --- | --- | --- |
| cluster 2 - cluster 1 | 0.02 | 0.04 | -0.12 0.08 | -0.57 | 0.98 |
| cluster 3 - cluster 1 | 0.03 | 0.04 | -0.14 0.09 | -0.69 | 0.96 |
| cluster 4 - cluster 1 | -0.06 | 0.03 | -0.03 0.14 | 1.77 | 0.40 |
| cluster 5 - cluster 1 | -0.07 | 0.04 | -0.04 0.18 | 1.83 | 0.36 |
| cluster 3 - cluster 2 | 8.43×10^-3^ | 0.04 | -0.12 0.10 | -0.22 | 1.00 |
| cluster 4 - cluster 2 | -0.08 | 0.03 | -1.46×10^-3^ 0.15 | 2.72 | 0.06* |
| cluster 5 - cluster 2 | -0.09 | 0.04 | -8.43×10^-3^ 0.19 | 2.54 | 0.09* |
| cluster 4 - cluster 3 | -0.08 | 0.03 | -0.01 0.18 | 2.41 | 0.12 |
| cluster 5 - cluster 3 | -0.10 | 0.04 | -0.02 0.22 | 2.40 | 0.12 |
| cluster 5 - cluster 4 | -0.02 | 0.03 | -0.07 0.11 | 0.49 | 0.99 |

**p* $\leq$ .05.

# **Table A22 |** Pairwise Comparisons of Positive Recall Bias Across Combined Clusters

| Comparison | Mean difference | SE | 95% CI | t | p |
| --- | --- | --- | --- | --- | --- |
| cluster 2 - cluster 1 | 0.011 | 0.036 | -0.155 0.108 | -0.306 | 1.000 |
| cluster 3 - cluster 1 | 0.017 | 0.038 | -0.218 0.057 | -0.459 | 0.999 |
| cluster 4 - cluster 1 | 0.006 | 0.030 | -0.173 0.046 | -0.190 | 1.000 |
| cluster 5 - cluster 1 | -0.054 | 0.030 | -0.215 0.009 | 1.764 | 0.573 |
| cluster 6 - cluster 1 | -0.020 | 0.044 | -0.261 0.058 | 0.453 | 0.999 |
| cluster 7 - cluster 1 | -0.065 | 0.029 | -0.215 -0.001 | 2.222 | 0.288 |
| cluster 3 - cluster 2 | 0.006 | 0.042 | -0.208 0.094 | -0.151 | 1.000 |
| cluster 4 - cluster 2 | -0.005 | 0.035 | -0.166 0.086 | 0.153 | 1.000 |
| cluster 5 - cluster 2 | -0.065 | 0.035 | -0.208 0.048 | 1.834 | 0.526 |
| cluster 6 - cluster 2 | -0.031 | 0.047 | -0.249 0.093 | 0.653 | 0.995 |
| cluster 7 - cluster 2 | -0.076 | 0.034 | -0.208 0.039 | 2.216 | 0.291 |
| cluster 4 - cluster 3 | -0.012 | 0.036 | -0.115 0.149 | 0.319 | 1.000 |
| cluster 5 - cluster 3 | -0.071 | 0.037 | -0.157 0.111 | 1.921 | 0.468 |
| cluster 6 - cluster 3 | -0.037 | 0.049 | -0.197 0.155 | 0.765 | 0.988 |
| cluster 7 - cluster 3 | -0.082 | 0.036 | -0.158 0.102 | 2.285 | 0.256 |
| cluster 5 - cluster 4 | -0.059 | 0.029 | -0.145 0.066 | 2.046 | 0.389 |
| cluster 6 - cluster 4 | -0.025 | 0.043 | -0.193 0.117 | 0.596 | 0.997 |
| cluster 7 - cluster 4 | -0.070 | 0.028 | -0.144 0.055 | 2.548 | 0.147 |
| cluster 6 - cluster 5 | 0.034 | 0.043 | -0.155 0.158 | -0.786 | 0.986 |
| cluster 7 - cluster 5 | -0.011 | 0.028 | -0.107 0.097 | 0.382 | 1.000 |
| cluster 7 - cluster 6 | -0.045 | 0.042 | -0.160 0.146 | 1.060 | 0.939 |

# **Table A23 |** Pairwise Comparisons of Difference between Negative and Positive Recall Bias Across Combined Clusters

| Comparison | Mean difference | SE | 95% CI | t | p |
| --- | --- | --- | --- | --- | --- |
| cluster 2 - cluster 1 | 0.023 | 0.044 | -0.155 0.108 | -0.530 | 0.998 |
| cluster 3 - cluster 1 | 0.080 | 0.046 | -0.218 0.057 | -1.740 | 0.590 |
| cluster 4 - cluster 1 | 0.064 | 0.037 | -0.173 0.046 | -1.724 | 0.601 |
| cluster 5 - cluster 1 | 0.103 | 0.038 | -0.215 0.009 | -2.741 | 0.093 |
| cluster 6 - cluster 1 | 0.101 | 0.054 | -0.261 0.058 | -1.891 | 0.489 |
| cluster 7 - cluster 1 | 0.108 | 0.036 | -0.215 -0.001 | -3.010 | 0.045* |
| cluster 3 - cluster 2 | 0.057 | 0.051 | -0.208 0.094 | -1.124 | 0.920 |
| cluster 4 - cluster 2 | 0.040 | 0.042 | -0.166 0.086 | -0.949 | 0.964 |
| cluster 5 - cluster 2 | 0.080 | 0.043 | -0.208 0.048 | -1.855 | 0.512 |
| cluster 6 - cluster 2 | 0.078 | 0.058 | -0.249 0.093 | -1.356 | 0.825 |
| cluster 7 - cluster 2 | 0.085 | 0.042 | -0.208 0.039 | -2.042 | 0.391 |
| cluster 4 - cluster 3 | -0.017 | 0.044 | -0.115 0.149 | 0.379 | 1.000 |
| cluster 5 - cluster 3 | 0.023 | 0.045 | -0.157 0.111 | -0.506 | 0.999 |
| cluster 6 - cluster 3 | 0.021 | 0.059 | -0.197 0.155 | -0.355 | 1.000 |
| cluster 7 - cluster 3 | 0.028 | 0.044 | -0.158 0.102 | -0.637 | 0.996 |
| cluster 5 - cluster 4 | 0.040 | 0.035 | -0.145 0.066 | -1.120 | 0.922 |
| cluster 6 - cluster 4 | 0.038 | 0.052 | -0.193 0.117 | -0.726 | 0.991 |
| cluster 7 - cluster 4 | 0.045 | 0.034 | -0.144 0.055 | -1.329 | 0.838 |
| cluster 6 - cluster 5 | -0.002 | 0.053 | -0.155 0.158 | 0.034 | 1.000 |
| cluster 7 - cluster 5 | 0.005 | 0.034 | -0.107 0.097 | -0.145 | 1.000 |
| cluster 7 - cluster 6 | 0.007 | 0.051 | -0.160 0.146 | -0.132 | 1.000 |

**p* $\leq$ .05.
