# Supplementary material for: Transdiagnostic clustering of self-schema from self-referential judgements identifies subtypes of healthy personality and depression
Source: Front Neuroinform. 2024 Jan 11;17:1244347. doi: 10.3389/fninf.2023.1244347 (PMC10808829; doi:10.3389/fninf.2023.1244347)
Supplement: Supplementary file 9 [file Table_9.DOCX]

***Supplementary Material***

# **Table A24 |** Pairwise Comparisons of Depressive Symptoms Across Clinical Clusters

| Comparison | Mean difference | SE | 95% CI | t | p |
| --- | --- | --- | --- | --- | --- |
| cluster 2 - cluster 1 | -6.04 | 4.20 | -17.69 - 5.61 | -1.44 | 0.60 |
| cluster 3 - cluster 1 | -5.95 | 4.88 | -19.49 - 7.59 | -1.22 | 0.74 |
| cluster 4 - cluster 1 | -4.74 | 3.73 | -15.08 - 5.61 | -1.27 | 0.70 |
| cluster 5 - cluster 1 | -17.41 | 4.62 | -30.21 - -4.61 | -3.77 | 0.0023** |
| cluster 3 - cluster 2 | 0.0087 | 4.57 | -12.57 - 12.74 | 0.019 | 1.00 |
| cluster 4 - cluster 2 | 1.30 | 3.30 | -7.86 - 10.45 | 0.39 | 0.99 |
| cluster 5 - cluster 2 | -11.38 | 4.28 | -23.24 - 0.49 | -2.66 | 0.06 |
| cluster 4 - cluster 3 | 1.21 | 4.14 | -10.25 - 12.67 | 0.29 | 1.00 |
| cluster 5 - cluster 3 | -11.46 | 4.95 | -25.18 - 2.26 | -2.31 | 0.14 |
| cluster 5 - cluster 4 | -12.67 | 3.82 | -23.26 - -2.09 | -3.32 | 0.01* |

# **p* $\leq$ *.05. **p* $\leq$ *.01.*

# **Table A25 |** Pairwise Comparisons of Depressive Symptoms Across Combined Clusters

| Comparison | Mean difference | SE | 95% CI | t | p |
| --- | --- | --- | --- | --- | --- |
| cluster 2 - cluster 1 | 17.30 | 3.37 | -27.33 - 7.27 | -5.13 | < .001*** |
| cluster 3 - cluster 1 | 19.23 | 3.53 | -29.72 - 8.74 | -5.45 | < .001*** |
| cluster 4 - cluster 1 | -0.38 | 2.80 | -7.94 - 8.7 | 0.13 | 1 |
| cluster 5 - cluster 1 | 9.75 | 2.86 | -18.25 -1.25 | -3.41 | 0.01* |
| cluster 6 - cluster 1 | 9.77 | 4.10 | -21.97 2.43 | -2.38 | 0.21 |
| cluster 7 - cluster 1 | 20.54 | 2.72 | -28.64 - 12.44 | -7.54 | < .001*** |
| cluster 3 - cluster 2 | 1.93 | 3.90 | -13.54 9.67 | -0.5 | 1 |
| cluster 4 - cluster 2 | -17.67 | 3.26 | 7.98 27.37 | 5.42 | < .001*** |
| cluster 5 - cluster 2 | -7.55 | 3.31 | -2.3 17.4 | 2.28 | 0.26 |
| cluster 6 - cluster 2 | -7.53 | 4.43 | -5.65 20.7 | 1.7 | 0.62 |
| cluster 7 - cluster 2 | 3.24 | 3.20 | -12.75 6.26 | -1.02 | 0.95 |
| cluster 4 - cluster 3 | -19.61 | 3.42 | 9.44 29.78 | 5.74 | < .001*** |
| cluster 5 - cluster 3 | -9.48 | 3.47 | -0.83 19.80 | 2.74 | 0.09 |
| cluster 6 - cluster 3 | -9.46 | 4.55 | -4.07 22.98 | 2.08 | 0.37 |
| cluster 7 - cluster 3 | 1.31 | 3.36 | -11.30 8.68 | -0.39 | 1 |
| cluster 5 - cluster 4 | 10.12 | 2.72 | -18.23 -2.02 | -3.72 | 4.69×10-3** |
| cluster 6 - cluster 4 | 10.15 | 4.01 | -22.07 1.78 | -2.53 | 0.15 |
| cluster 7 - cluster 4 | 20.92 | 2.58 | -28.6 -13.23 | -8.1 | < .001*** |
| cluster 6 - cluster 5 | 0.03 | 4.05 | -12.08 12.02 | -6.33×10-3 | 1 |
| cluster 7 - cluster 5 | 10.79 | 2.65 | -18.67 -2.92 | -4.08 | 1.22×10-3** |
| cluster 7 - cluster 6 | 10.77 | 3.96 | -22.54 1 | -2.72 | 0.1 |

**p* $\leq$ .05. ***p* $\leq$ .01. ****p* $\leq$ .001.
