# Supplementary material for: Transdiagnostic clustering of self-schema from self-referential judgements identifies subtypes of healthy personality and depression
Source: Front Neuroinform. 2024 Jan 11;17:1244347. doi: 10.3389/fninf.2023.1244347 (PMC10808829; doi:10.3389/fninf.2023.1244347)
Supplement: Supplementary file 10 [file Table_10.DOCX]

***Supplementary Material***

**TABLE A3 |** Hierarchical Multiple Regression of the Effect of Number of Negative Words Endorsed On Depressive Symptoms Over and Above Age and Gender in Clinical Sample

|  | **Model 1**  **DV = Depressive Symptoms** | | | | | **Model 2**  **DV = Depressive Symptoms** | | | | |
| --- | --- | --- | --- | --- | --- | --- | --- | --- | --- | --- |
| *Predictors* | *B* | *β* | *CI* | *Standardized CI* | *p* | *B* | *β* | *CI* | *Standardized CI* | *p* |
| (Intercept) | 27.44 | 0.00 | 16.31 - 38.57 | -0.18 - 0.18 | **<0.001** | 14.78 | 0.00 | 1.74 - 27.83 | -0.17 - 0.17 | **0.027** |
| Age | -0.03 | -0.02 | -0.26 - 0.20 | -0.21 - 0.16 | 0.804 | 0.03 | 0.03 | -0.19 - 0.25 | -0.15 - 0.20 | 0.780 |
| Gender | 3.16 | 0.11 | -1.90- 8.23 | -0.07 - 0.30 | 0.218 | 2.72 | 0.10 | -2.15 - 7.58 | -0.08 - 0.27 | 0.271 |
| Number of negative words endorsed |  | | | | | 0.84 | 0.30 | 0.34 - 1.34 | 0.12 - 0.48 | **0.001** |
| Observations | 119 |  |  |  |  | 119 |  |  |  |  |
| R^2^ / R^2^ adjusted | 0.014 / -0.003 | |  |  |  | 0.101 / 0.078 | |  |  |  |
| Total F (DFn, DFd) | 0.82 (2, 116) | |  |  |  | 4.31 (3, 115) | |  |  |  |
| Note. Δ*R^2^* from model 1 to model 2 = 0.087, Δ*F* from model 1 to model 2 = 11.17. Significant p-values are bolded | | | | | | | | | | |

**TABLE A4 |** Hierarchical Multiple Regression of the Effect of Number of Negative Words Endorsed On Depressive Symptoms Over and Above Age and Gender in Non-clinical Sample

|  | Model 1  DV=Depressive Symptoms | | | | | Model 2  DV=Depressive Symptoms | | | | |
| --- | --- | --- | --- | --- | --- | --- | --- | --- | --- | --- |
| *Predictors* | B | *β* | *CI* | *standardized CI* | *p* | *B* | *β* | *CI* | *standardized CI* | *p* |
| (Intercept) | 12.83 | -0.00 | -4.43 – 30.09 | -0.19 – 0.19 | 0.144 | 9.39 | -0.00 | -7.65 – 26.43 | -0.18 – 0.18 | 0.277 |
| Age | 0.04 | 0.01 | -0.55 – 0.63 | -0.18 – 0.20 | 0.887 | 0.02 | 0.01 | -0.56 – 0.60 | -0.18 – 0.19 | 0.942 |
| Gender | 0.42 | 0.02 | -4.19 – 5.04 | -0.17 – 0.21 | 0.856 | 0.14 | 0.01 | -4.36 – 4.65 | -0.18 – 0.19 | 0.950 |
| Number of negative words endorsed |  |  |  |  |  | 0.59 | 0.24 | 0.14 – 1.03 | 0.06 – 0.42 | **0.011** |
| Observations | 115 | |  |  |  | 115 | |  |  |  |
| R^2^ / R^2^ adjusted | 0.000 / -0.017 | |  |  |  | 0.058 / 0.032 | |  |  |  |
| Total F (DFn, DFd) | 0.023 (2, 112) | |  |  |  | 2.27 (3, 111) | |  |  |  |

Note. Δ*R^2^* from model 1 to model 2 = 0.058, Δ*F* from model 1 to model 2 = 6.75. Significant p-values are bolded.

**TABLE A5 |** Hierarchical Multiple Regression of the Effect of Number of Negative Words Endorsed On Depressive Symptoms Over and Above Age and Gender in Overall Sample

|  | **Model 1**  **DV = Depressive Symptoms** | | | | | **Model 2**  **DV = Depressive Symptoms** | | | | |
| --- | --- | --- | --- | --- | --- | --- | --- | --- | --- | --- |
| *Predictors* | *B* | *β* | *CI* | *Standardized CI* | *p* | *B* | *β* | *CI* | *Standardized CI* | *p* |
| (Intercept) | 12.50 | -0.00 | 3.19 - 21.80 | -0.13 - 0.13 | **0.009** | 2.09 | -0.00 | -6.55 - 10.74 | -0.11 - 0.11 | 0.634 |
| Age | 0.38 | 0.23 | 0.17 - 0.58 | 0.10 - 0.36 | **<0.001** | 0.26 | 0.16 | 0.08 - 0.28 | 0.05 - 0.28 | **0.006** |
| Gender | -0.04 | -0.00 | -3.97 - 3.89 | -0.13 - 0.13 | 0.982 | 0.28 | 0.01 | -3.21 - 3.76 | -0.10 - 0.12 | 0.875 |
| Number of negative words endorsed |  | | | | | 1.23 | 0.46 | 0.93 - 1.54 | 0.35 - 0.57 | **<0.001** |
| Observations | 234 |  |  |  |  | 234 |  |  |  |  |
| R^2^ / R^2^ adjusted | 0.053 / 0.044 | |  |  |  | 0.258 / 0.248 | |  |  |  |
| Total F (DFn, DFd) | 6.42 (2, 231) | |  |  |  | 26.68 (3, 230) | |  |  |  |
| Note. Δ*R^2^* from model 1 to model 2 = 0.205, Δ*F* from model 1 to model 2 = 63.71. Significant p-values are bolded. | | | | | | | | | | |

## **TABLE A6 |** Hierarchical Multiple Regression of the Effect of Number of Negative Words Endorsed On Depressive Symptoms Over and Above Age, Gender, and Group in Overall Sample

|  | **Model 1**  **DV = Depressive Symptoms** | | | | | **Model 2**  **DV = Depressive Symptoms** | | | | |
| --- | --- | --- | --- | --- | --- | --- | --- | --- | --- | --- |
| *Predictors* | *B* | *β* | *CI* | *Standardized CI* | *p* | *B* | *β* | *CI* | *Standardized CI* | *p* |
| (Intercept) | 11.99 | -0.00 | 3.99 - 19.99 | -0.11 - 0.11 | **0.003** | 6.05 | -0.00 | -2.14 - 14.24 | -0.10 - 0.10 | 0.147 |
| Age | -0.02 | -0.01 | -0.22 - 0.18 | -0.13 - 0.11 | **0.828** | 0.02 | 0.01 | -0.17 - 0.21 | -0.10 - 0.13 | 0.823 |
| Gender | 1.86 | 0.06 | -1.54 - 5.27 | -0.05 - 0.17 | 0.282 | 1.53 | 0.05 | -1.76 - 4.81 | -0.06 - 0.16 | 0.360 |
| Group | 17.19 | 0.56 | 13.46 - 20.91 | 0.44 - 0.69 | **<0.001** | 12.47 | 0.41 | 8.27 - 16.67 | 0.27 - 0.55 | **<0.001** |
| Number of negative words endorsed |  | | | | | 0.72 | 0.27 | 0.39 - 1.05 | 0.14 - 0.39 | **<0.001** |
| Observations | 234 |  |  |  |  | 234 |  |  |  |  |
| R^2^ / R^2^ adjusted | 0.303 / 0.294 | |  |  |  | 0.355 / 0.343 | |  |  |  |
| Total F (DFn, DFd) | 33.38 (3, 230) | |  |  |  | 31.46 (4, 229) | |  |  |  |
| Note. Δ*R^2^* from model 1 to model 2 = 0.052, Δ*F* from model 1 to model 2 = 18.23. Significant p-values are bolded. | | | | | | | | | | |

**TABLE A7 |** Hierarchical Multiple Regression of the Effect of Number of Positive Words Endorsed On Depressive Symptoms Over and Above Age, Gender, and Group in Overall Sample

|  | Model 1  DV=Depressive Symptoms | | | | | Model 2  DV=Depressive Symptoms | | | | |
| --- | --- | --- | --- | --- | --- | --- | --- | --- | --- | --- |
| *Predictors* | B | *β* | *CI* | *standardized CI* | *p* | *B* | *β* | *CI* | *standardized CI* | *p* |
| (Intercept) | 11.99 | -0.00 | 3.99 – 19.99 | -0.11 – 0.11 | **0.003** | 16.34 | -0.00 | 7.28 – 25.40 | -0.11 – 0.11 | **<0.001** |
| Age | -0.02 | -0.01 | -0.22 – 0.18 | -0.13 – 0.11 | 0.828 | -0.02 | -0.01 | -0.21 – 0.18 | -0.13 – 0.11 | 0.863 |
| Gender | 1.86 | 0.06 | -1.54 – 5.27 | -0.05 – 0.17 | 0.282 | 2.05 | 0.07 | -1.34 – 5.43 | -0.04 – 0.18 | 0.235 |
| Group | 17.19 | 0.56 | 13.46 – 20.91 | 0.44 – 0.69 | **<0.001** | 15.83 | 0.52 | 11.89 – 19.77 | 0.39 – 0.65 | **<0.001** |
| Number of positive words endorsed |  |  |  |  |  | -0.36 | -0.12 | -0.73 – 0.00 | -0.23 – 0.00 | **0.050** |
| Observations | 234 | |  |  |  | 234 | |  |  |  |
| R^2^ / R^2^ adjusted | 0.303 / 0.294 | |  |  |  | 0.315 / 0.303 | |  |  |  |
| Total F (DFn, DFd) | 33.38 (3, 230) | |  |  |  | 26.32 (4, 229) | |  |  |  |

Note. Δ*R^2^* from model 1 to model 2 = 0.012, Δ*F* from model 1 to model 2 = 3.89. Significant p-values are bolded.

**TABLE A8 |** Hierarchical Multiple Regression of the Effect of Negative RT Bias On Depressive Symptoms Over and Above Age, Gender, and Group in Overall Sample

|  | **Model 1**  **DV = Depressive Symptoms** | | | | | **Model 2**  **DV = Depressive Symptoms** | | | | |
| --- | --- | --- | --- | --- | --- | --- | --- | --- | --- | --- |
| *Predictors* | *B* | *β* | *CI* | *Standardized CI* | *p* | *B* | *β* | *CI* | *Standardized CI* | *p* |
| (Intercept) | 11.78 | 0.00 | 3.44 - 20.12 | -0.11 - 0.11 | **0.006** | 12.01 | 0.00 | 3.73 - 20.28 | -0.11 - 0.11 | **0.005** |
| Age | -0.02 | -0.01 | -0.23 - 0.18 | -0.14 - 0.11 | 0.831 | -0.02 | -0.01 | -0.22 - 0.18 | -0.14 - 0.11 | 0.837 |
| Gender | 2.07 | 0.07 | -1.47 - 5.61 | -0.05 - 0.18 | 0.250 | 1.89 | 0.06 | -1.63 - 5.41 | -0.05 - 0.17 | 0.291 |
| Group | 17.00 | 0.55 | 13.14 - 20.86 | 0.43 - 0.68 | **<0.001** | 16.00 | 0.52 | 12.06 - 19.94 | 0.39 - 0.65 | **<0.001** |
| Negative RT Bias |  | | | | | -4.0 | -0.12 | -7.73 - -0.27 | -0.24 - -0.01 | **0.036** |
| Observations | 234 |  |  |  |  | 234 |  |  |  |  |
| R^2^ / R^2^ adjusted | 0.292 / 0.282 | |  |  |  | 0.306 / 0.292 | |  |  |  |
| Total F (DFn, DFd) | 30.24 (3, 220) | |  |  |  | 24.16 (4, 219) | |  |  |  |
| Note. Δ*R^2^* from model 1 to model 2 = 0.014, Δ*F* from model 1 to model 2 = 4.47. Significant p-values are bolded. | | | | | | | | | | |

**TABLE A9 |** Hierarchical Multiple Regression of the Effect of Positive RT Bias On Depressive Symptoms Over and Above Age, Gender, and Group in Overall Sample

|  | **Model 1**  **DV = Depressive Symptoms** | | | | | **Model 2**  **DV = Depressive Symptoms** | | | | |
| --- | --- | --- | --- | --- | --- | --- | --- | --- | --- | --- |
| *Predictors* | *B* | *β* | *CI* | *Standardized CI* | *p* | *B* | *β* | *CI* | *Standardized CI* | *p* |
| (Intercept) | 12.64 | 0.00 | 4.20 - 21.07 | -0.11 - 0.11 | **0.003** | 13.25 | 0.00 | 4.72 - 21.79 | -0.11 - 0.11 | **0.002** |
| Age | -0.01 | -0.00 | -0.21 - 0.20 | -0.13 - 0.12 | 0.958 | -0.01 | -0.01 | -0.22 - 0.20 | -0.14 - 0.12 | 0.903 |
| Gender | 1.49 | 0.05 | -2.13 - 5.11 | -0.07 - 0.16 | 0.418 | 1.47 | 0.05 | -2.15 - 5.09 | -0.07 - 0.16 | 0.426 |
| Group | 16.69 | 0.54 | 12.73 - 20.64 | 0.41 - 0.67 | **<0.001** | 16.49 | 0.54 | 12.52 - 20.47 | 0.41 - 0.67 | **<0.001** |
| Positive RT Bias |  | | | | | 2.36 | 0.06 | -2.56 - 7.29 | -0.06 - 0.17 | 0.345 |
| Observations | 217 |  |  |  |  | 217 |  |  |  |  |
| R^2^ / R^2^ adjusted | 0.286 / 0.276 | |  |  |  | 0.289 / 0.275 | |  |  |  |
| Total F (DFn, DFd) | 28.39 (3, 213) | |  |  |  | 21.5 (4, 212) | |  |  |  |
| Note. Δ*R^2^* from model 1 to model 2 = 0.003, Δ*F* from model 1 to model 2 = 0.896. Significant p-values are bolded. | | | | | | | | | | |

**TABLE A10 |** Hierarchical Multiple Regression of the Effect of Negative Recall Bias On Depressive Symptoms Over and Above Age, Gender, and Group in Overall Sample

|  | Model 1  DV=Depressive Symptoms | | | | | Model 2  DV=Depressive Symptoms | | | | |
| --- | --- | --- | --- | --- | --- | --- | --- | --- | --- | --- |
| *Predictors* | B | *β* | *CI* | *standardized CI* | *p* | *B* | *β* | *CI* | *standardized CI* | *p* |
| (Intercept) | 12.67 | 0.00 | 3.59 – 21.76 | -0.12 – 0.12 | **0.006** | 9.42 | 0.00 | 0.07 – 18.76 | -0.12 – 0.12 | **0.048** |
| Age | -0.05 | -0.03 | -0.29 – 0.19 | -0.16 – 0.10 | 0.669 | -0.02 | -0.01 | -0.25 – 0.22 | -0.14 – 0.12 | 0.886 |
| Gender | 2.02 | 0.06 | -1.69 – 5.72 | -0.05 – 0.18 | 0.285 | 2.27 | 0.07 | -1.40 – 5.94 | -0.04 – 0.19 | 0.224 |
| Group | 17.08 | 0.56 | 13.04 – 21.12 | 0.42 – 0.69 | **<0.001** | 15.26 | 0.50 | 11.02 – 19.50 | 0.36 – 0.63 | **<0.001** |
| Negative Recall Bias |  |  |  |  |  | 6.73 | 0.15 | 1.35 – 12.11 | 0.03 – 0.28 | **0.014** |
| Observations | 207 | |  |  |  | 207 | |  |  |  |
| R^2^ / R^2^ adjusted | 0.287 / 0.277 | | |  |  | 0.308 / 0.294 | |  |  |  |
| Total F (DFn, DFd) | 27.27 (3, 203) | | |  |  | 22.49 (4, 202) | |  |  |  |

Note. Δ*R^2^* from model 1 to model 2 = 0.021, Δ*F* from model 1 to model 2 = 6.09. Significant p-values are bolded.
