# Supplementary figures and images for: Transdiagnostic clustering of self-schema from self-referential judgements identifies subtypes of healthy personality and depression
Source: Front Neuroinform. 2024 Jan 11;17:1244347. doi: 10.3389/fninf.2023.1244347 (PMC10808829; doi:10.3389/fninf.2023.1244347)

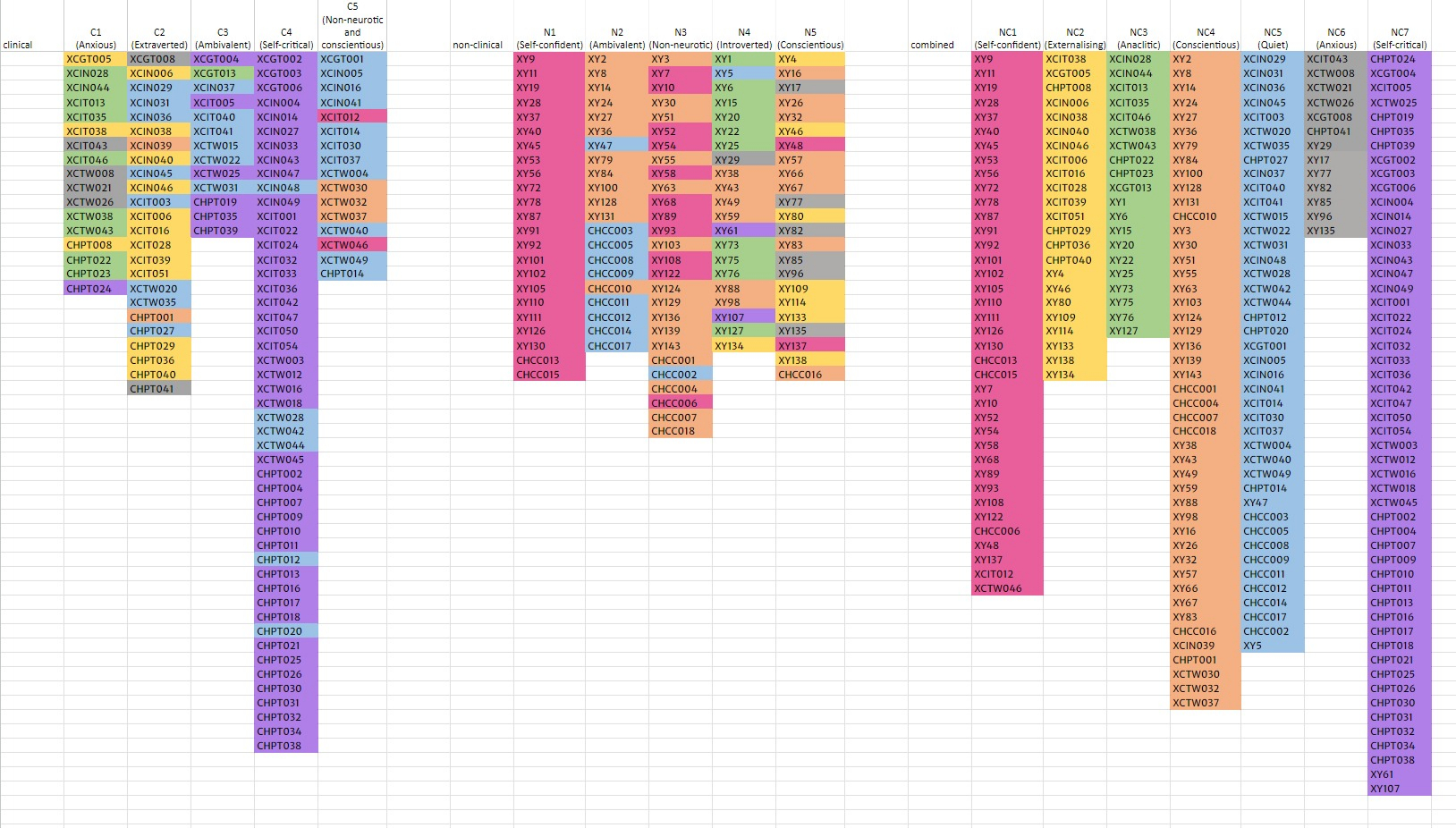

Supplement: Supplementary file 11 [file Image_1.JPEG]

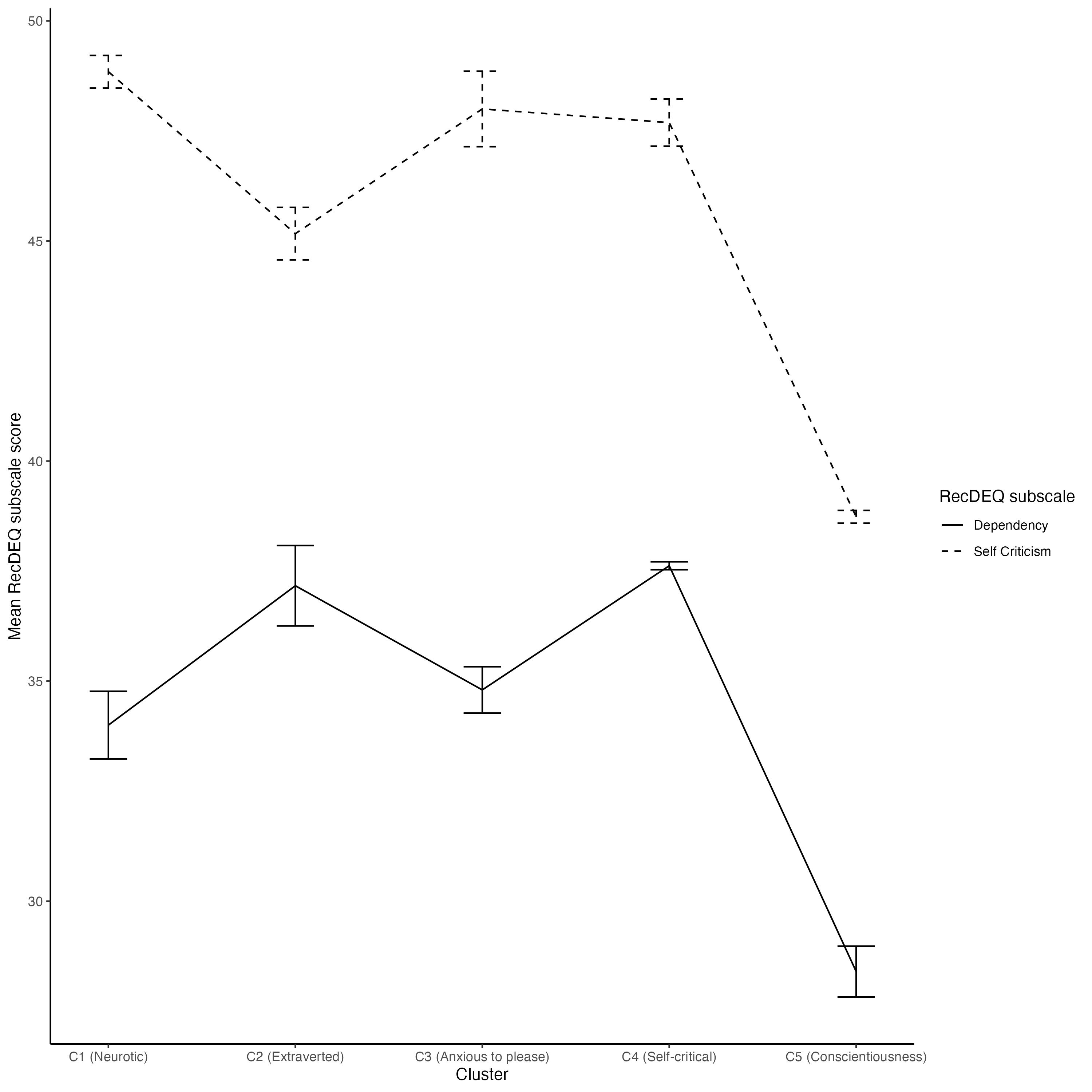

Supplement: Supplementary file 12 [file Image_2.JPEG]
